# Predator selection on phenotypic variability of cryptic and aposematic moths

**DOI:** 10.1038/s41467-024-45329-5

**Published:** 2024-02-23

**Authors:** Ossi Nokelainen, Sanni A. Silvasti, Sharon Y. Strauss, Niklas Wahlberg, Johanna Mappes

**Affiliations:** 1 Organismal and Evolutionary Biology Research Programme, Faculty of Biological and Environmental Sciences, University of Helsinki, Viikki Biocenter 3, P.O. Box 65, 40014 Helsinki, Finland; 2 Department of Biological and Environmental Science, University of Jyväskylä, P.O. Box 35, FI-40014 Jyväskylä, Finland; 3 Open Science Centre, University of Jyväskylä, P.O. Box 35, FI-40014 Jyväskylä, Finland; 4 School of Natural Sciences, Macquarie University, Sydney, NSW 2109 Australia; 5 Department of Evolution and Ecology, University of California at Davis, 2320 Storer Hall, One Shields Avenue, Davis, CA 95616 USA; 6 Wissenschaftskolleg zu Berlin, Wallotstrasse 19, Berlin, 14193 Germany; 7 Department of Biology, Lund University, Sölvegatan 37, SE-223 62, Lund, Sweden

**Keywords:** Behavioural ecology, Coevolution, Evolutionary ecology

## Abstract

Natural selection generally favours phenotypic variability in camouflaged organisms, whereas aposematic organisms are expected to evolve a more uniform warning coloration. However, no comprehensive analysis of the phenotypic consequences of predator selection in aposematic and cryptic species exists. Using state-of-the-art image analysis, we examine 2800 wing images of 82 moth species accessed via three online museum databases. We test whether anti-predator strategy (i.e., camouflage or aposematism) explains intraspecific variation in wing colour and pattern across northern hemisphere moths. In addition, we test two mutually non-exclusive, ecological hypotheses to explain variation in colour pattern: diel-activity or dietary-niche. In this work, taking into account phylogenetic relationships, moth phenotypic variability is best explained by anti-predator strategy with camouflaged moths being more variable in wing patterning than aposematic species.

## Abstract (web summary)

Selection is expected to act differently on aposematic and cryptic species. Analysis of wing images revealed that camouflaged moths exhibit higher wing pattern variability than aposematic moths, supporting the theory that camouflaged species display more variability, consistent with anti-predator strategy.

## Introduction

Visual predators are known to exert selection that drives prey to evolve a wealth of appearances^1–3^. For instance, in moths (Lepidoptera), which are commonly preyed on by bats during night and birds during day^4,5^, predation has led to elaborate camouflage types that help ‘cryptic’ (i.e., camouflaged phenotypes that reduce detection) moths conceal themselves while resting during daytime^5–7^. Some moths have evolved other defence strategies that have enabled them to become diurnally more active^8,9^. Many diurnal moths have evolved to sequester or synthesise chemical compounds that make them unprofitable for predators^10–12^. Such chemically defended species have conspicuous colours and patterns that function as warning signals for predators and are called aposematic^3,13^.

Current theory states that camouflaged organisms should be phenotypically more variable than aposematic ones^3,13^. Birds are important visual predators of both diurnal and nocturnal species of moths^5,14–16^. Although moths are also preyed upon by other predators (e.g., bats), such selection on is expected to act on traits other than colouration, as nocturnal bats use other cues^17^. Visual daytime predators hunting camouflaged prey may increase foraging efficiency by forming a search image of their prey that enables them to detect prey more easily in complex backgrounds^18,19^. Search images (as memory for prey types) are thought to trigger negative frequency-dependent selection by predators against the most common morphotype and lead to increased colour and pattern polymorphism in the population^18–23^. This expectation is supported in noctuid moths by studies that demonstrate that moth species with more variable colour patterns have higher fitness than less varying species^24,25^. Another mechanism generating variability in camouflaged prey could be if camouflage is required to blend into variable backgrounds (e.g., matching differently sized textures). Prey might be more variable as they are under weak selection to match a variety of backgrounds. This variability in part may result from an increase of rare alleles by genetic drift, rather than due to strict negative frequency-dependent selection^26,27^.

From the signal theory perspective, aposematic organisms are predicted to benefit from maximising their signal-to-noise ratio (i.e., prey being the ‘signal’ for the predator and ‘noise’ being the visual environment) by being conspicuously coloured and easily detectable from their surroundings^28–30^. Conspicuous warning colouration facilitates effective signal learning and helps to separate aposematic prey from more profitable prey types^3,31–37^. Warning signals are most effective when the signal does not vary, because predators can more easily learn one signal than many^38,39^. Thus, aposematic prey are repeatedly shown to be locally under positive frequency-dependent selection and under stabilising selection for uniform signals^26,27,40–45^. At the same time, there are many famous aposematic and Müllerian mimicry systems that are phenotypically variable and even stable polymorphism is common, which does not fit the classic paradigm^14,46–52^. More recently, it has even been argued that large phenotypic variation in aposematic organisms should be considered the new norm^53^. However, there are no data that would allow us to compare relative levels of intraspecific variability among aposematic and cryptic species.

Naturally, other aspects of ecology might also influence the evolution of colour pattern in Lepidoptera, and here we consider two explicitly: diel-activity and diet breadth. Diel-activity could be related to moth phenotypic variation as selection from visual predators is most important during the daytime, when nocturnal moths are resting on bark or leaves. For camouflaged prey, predation should favour the colours and patterns resembling resting locations, but there would be no a priori prediction of greater variation in colouration across diel-activity traits, as both nocturnal and diurnal moth species experience predation from diurnal birds. For aposematic species, we expect less variation in aposematic diurnal moths than aposematic nocturnal moths under positive frequency-dependent or stabilising selection. We assume that in low-light conditions it is more difficult for predators to perceive colour variation and thus that nocturnal aposematic moths afford more colour variation than diurnal aposematic moths. For the latter, daylight conditions should enhance the transmission of colour signals to predators, which can be assumed to select for warning signal stability. Dietary specialisation could also be linked to phenotypic variation. Monophagous specialists that rest on their hosts are expected to closely match the surface of their preferred host plant and selection might thus reduce pattern variation in monophagous species, whereas polyphagous species utilising different host plants might be expected to be more variable; we expect this to be true only for camouflaged species.

Also, fore- and hindwings may not be under the same selective pressures from visual predators and may evolve independently, if forewings cover the whole moth at rest, and hindwings either carry striking patterns for startle defences, or are invisible to predators and selection from them^54–56^. For camouflaged species, we predict more variation in forewings for the same reasons as argued above; also, there may be weak or no selection on hindwings from daytime visual predators, as forewings typically cover the body and hindwings during rest^57^. For aposematic moth species, we predict less intraspecific variation than camouflaged species for both fore- and hindwings; aposematic species should be under stabilising selection to present consistent signals to predators and as aposematic species often rest with their hindwings partially exposed, thus exposing both wings to stabilising selection from predators^58–62^. Thus, we might expect fore- and hindwings to show different responses to selection^56^.

**Fig. 1:**
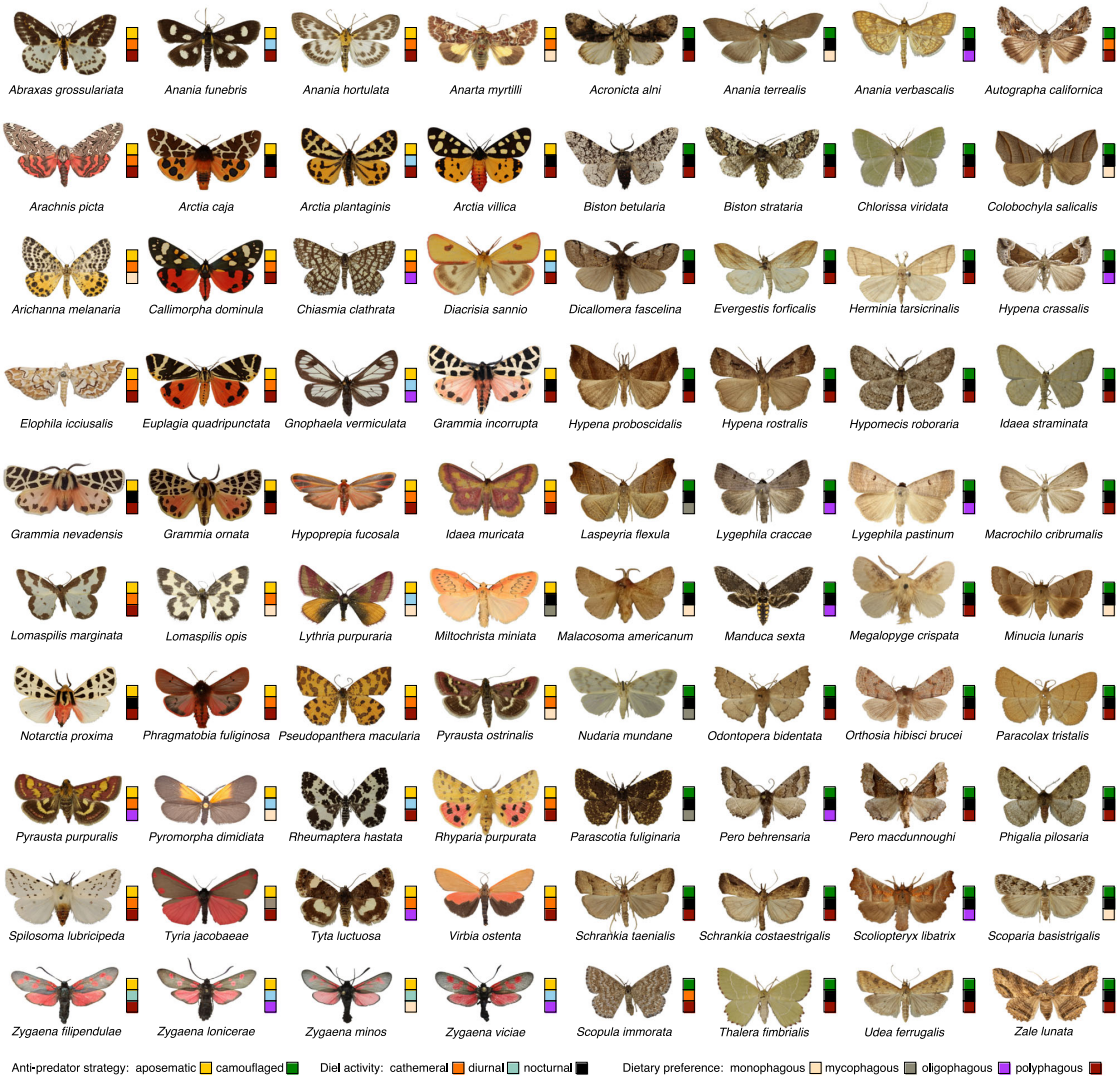
The species assembly of this work illustrating between-species differences in moth appearance. The moth images exemplify interspecific phenotypic variation across moth anti-predator strategies, diel-activity and dietary preference as flagged by the legend. Images are not normalised nor in actual scale. The first half on the left side shows species that are considered aposematic in literature and the second half on the right shows species that are broadly considered camouflaged. Two species are not shown for artistic reasons.

In order to evaluate expectations of relative intraspecific phenotypic variability in moths, we examined 2800 wing images of 82 moth species from eight moth families accessed via digitised online collections from natural history museums and universities across the northern hemisphere (Fig. 1). Adult moths are an ideal group for this analysis because they chiefly use pheromones in intraspecific communication^63^ and thus, sexual selection is a less important force contributing to colouration or patterning of moths. Colour and pattern phenotypes were quantified from collection images with the Multispectral Image Calibration and Analysis Toolbox and Quantitative Colour and Pattern Analysis plugin^64^ in ImageJ^65^. All colour and pattern metrics were tested for the fore and hindwings separately.

**Fig. 2:**
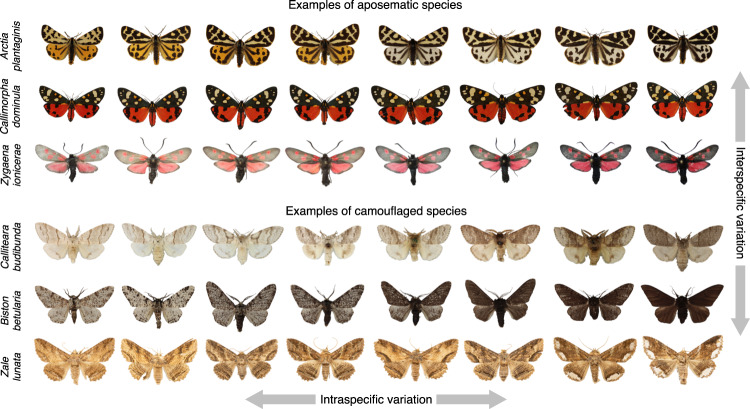
The moth assembly illustrating some examples of moth interspecific (rows) versus intraspecific (columns) phenotypic variation between aposematic and camouflage anti-predator strategies. Here, six species are shown. Notice how the marking size of the pattern characteristically varies more in camouflaged than aposematic species, albeit there is phenotypic variation across all moths. The figure highlights the care required to make helpful interpretations of phenotypic differences using between-species (i.e., interspecific) versus within-species (i.e., intraspecific) data, which characterise different biological hierarchies.

**Fig. 3:**
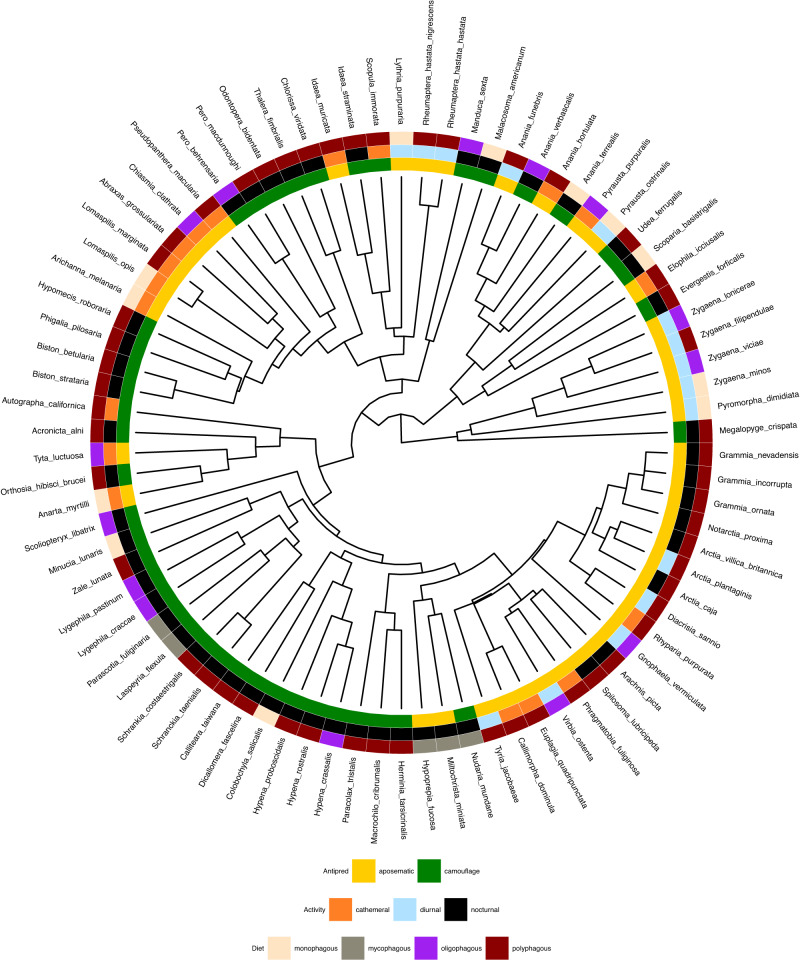
An ultrametric tree showing the phylogenetic relationships of the moth species used in this study. The legends annotate the alternative ecological hypotheses predicting their appearance as: anti-predator strategy (aposematism, camouflage), diel-activity (cathemeral, diurnal, nocturnal) and dietary preference (monophagous, mycophagous, oligophagous, polyphagous).

To assess within-species (i.e., intraspecific) variability (Fig. 2), we used the coefficient of variation [(cv), the standard deviation divided by the mean)]. We analysed the cv of standard colour components: hue, saturation and brightness; and of pattern traits: dominance, marking size and contrast (see methods). We asked whether anti-predator strategy (aposematism, camouflage), diet specialisation (monophagous, mycophagous, oligophagous, polyphagous) or active flying time [diurnal, cathemeral (dawn/dusk) or nocturnal] could explain differences in intraspecific variability while controlling for moth phylogeny (Fig. 3). In addition to intraspecific differences in the coefficient of variation in pattern and colour traits across colouration syndromes, we explored interspecific differences in mean values of these traits, even though they are not the crux of the hypotheses. We expect mean values to vary systematically between aposematic and camouflaged species. That is, mean contrast should be greater and colours more vivid in aposematic compared to camouflaged moths; the mean pattern size should also be larger in aposematic moths as large patterns send stronger signals and are easier to learn^66–68^. By using observational data comparing museum specimens, we cannot ascribe any mechanism underlying differences in intraspecific variability to for example, frequency-dependent selection versus relaxed selection. What we can do is to survey differences in the amount of intraspecific variability in aposematic and cryptic species and ask whether the results support or refute predictions above. All results were Holm–Bonferroni corrected for multiple comparisons (see 'Methods' for details) and effect sizes reported with Eta Squared (η2) statistics. In this work, taking into account phylogenetic relationships, moth phenotypic variability was best explained by anti-predator strategy. In line with classic theoretical predictions, our primary conclusion confirms that camouflaged moths show more variable wing patterning than aposematic.

## Results

### Differences in within-species variability

**Fig. 4:**
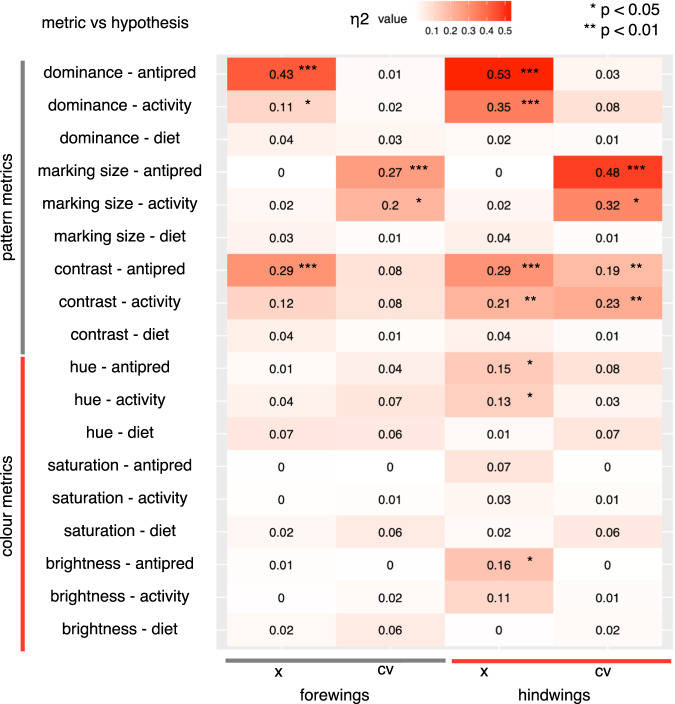
A heatmap plot that summarises significant associations of the moth colour and pattern as regards the alternative ecological hypotheses predicting their appearance. On the *x* axis values stands for forewing and hindwing, *x* for mean (i.e., interspecific variation) and cv for the coefficient of variation (i.e., intraspecific variation). On the *y* axis, different pattern and colour metrics are compared against their alternative hypotheses for moth phenotypic variability: anti-predator strategy, diel-activity and dietary-niche. The panel shows Eta Squared statistics for effect sizes and flags significant *P* values obtained from the phyloANOVA analysis (i.e., the higher η2-values and lower *P* values indicate higher statistical significance). All associated test statistics are based on two-tailed significance levels.

Out of the three ecological hypotheses (i.e., anti-predator strategy, diel-activity or dietary-niche), within-species phenotypic variability of moths was best explained by anti-predator strategy, less so by diel-activity patterns (see Fig. 4, effect sizes). In general, diel-activity predicted moth phenotypic variation in a similar way to anti-predator function, because most camouflaged moths were nocturnal (Fig. 3). We did not find any support for the dietary-niche hypothesis (diet breadth was never a significant predictor of moth phenotypic variation; Fig. 4). Noteworthily, moth size measured as wing area was not significantly explained by any of the three ecological predictors. Thus, the following results focus on differences between anti-predator strategy and diel-activity.

### Within-species variability in pattern traits

**Fig. 5:**
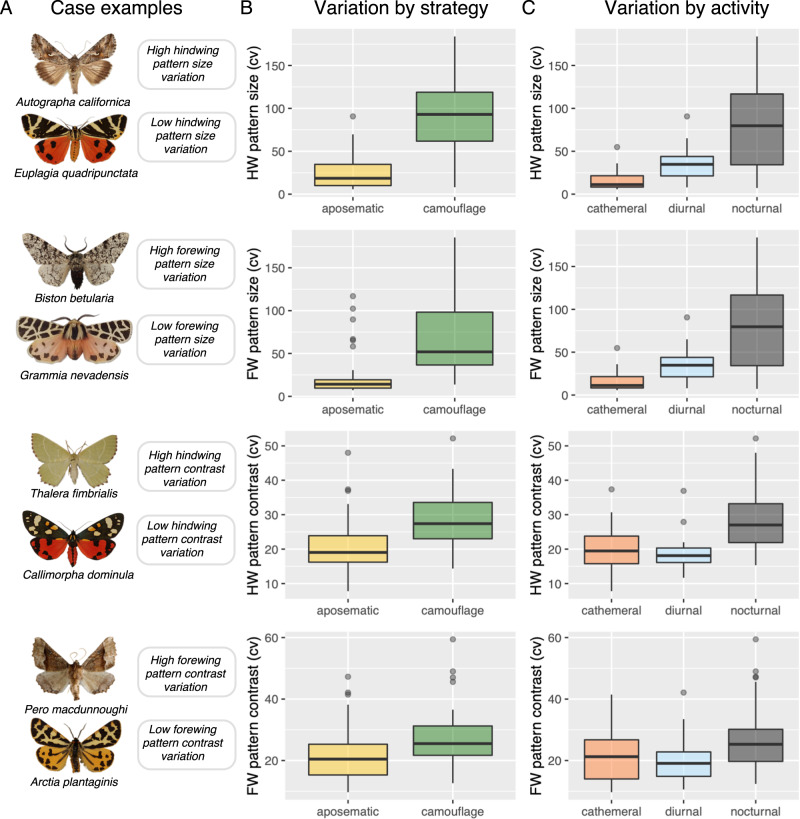
The primary variables of interest highlighting within-species phenotypic variability through variation of coefficients (cv). **A** shows case examples of different camouflaged and aposematic moth species and their characterisations. Boxplots are organised in descending order with respect to statistical significance and separate anti-predator strategy (**B**) and moth diel-activity (**C**). The boxplot shows minimum and maximum (whiskers), the median line and the interquartile range. Wing pattern values are shown for fore- and hindwings. **B**, **C** show the data of *n* = 2800 wing images of 82 species collated under respective anti-predator strategy (**B**) and diel-activity (**C**).

As predicted, camouflaged moths were more variable within species in wing pattern size in both forewings and hindwings (i.e., greater coefficient of variation) than aposematic species (Strategy—Marking size.cv. Forewing: *F* = 29.59, *P* = 0.001; hindwing: *F* = 72.91, *P* = 0.001). Pattern size variability was greater in hindwings than forewings (Fig. 5). Nocturnal moths were more variable within species in wing pattern size than diurnal or cathemeral (i.e., crepuscular or irregular behavioural activity) moths (Activity—Marking size.cv. Forewing: *F* = 10.14, *P* = 0.008; hindwing: *F* = 18.78, *P* = 0.001). Pattern contrast was also more variable in camouflaged than aposematic moths, but only in hindwings (Strategy—Contrast.cv. Forewing: *F* = 6.92, *P* = 0.096; hindwing: *F* = 18.69, *P* = 0.005). Nocturnal lineages of both aposematic and camouflaged species had more variable pattern contrast than diurnal species (Activity—Contrast.cv. Forewing: *F* = 3.20, *P* = 0.195; hindwing: *F* = 11.72, *P* = 0.003). Nocturnal moths also had greater variability in hindwing pattern contrast than cathemeral or diurnal moths (Fig. 4 and 5). Pattern dominance was not significant for anti-predator strategy (Strategy— Dominance.cv. Forewing: *F* = 1.03, *P* = 0.505; hindwing: *F* = 2.12, *P* = 0.342) or diel-activity (Activity—Dominance.cv. Forewing: *F* = 0.60, *P* = 0.712; hindwing: *F* = 3.66, *P* = 0.159).

### Within-species variability in colour traits

Counter to our predictions, we generally did not find evidence that within-species variability in colour traits is different between aposematic vs camouflaged species (Figs. 4 and 5), despite the fact that aposematic and camouflaged species differ markedly in mean values of colour traits (Supplementary Figs. 2–4). Albeit a statistically non-significant observation, aposematic moths show a little more variability in colour than camouflaged moths in hindwings, lumping across activity times (Strategy—Hue.cv. Forewing: *F* = 3.66, *P* = 0.229; hindwing: *F* = 6.68, *P* = 0.091).

### Across species, variation in colour and pattern traits

As expected, aposematic species had larger and more contrasting patterns than did camouflaged species; further results about the mean differences in colour and pattern metrics between species types are reported in supplementary materials (Supplementary Figs. 2–4).

## Discussion

We present evidence in support of the classic hypothesis that camouflaged organisms harbour more phenotypic variation than aposematic species across the moth phylogeny. Interestingly, our predictions on variability were supported only for pattern traits and not for colour traits; we found no differences in within-species variability in colour traits for any factor considered, even though mean hue, saturation and brightness of hindwings were greater in aposematic species, as expected. Selection appears to act more on variability in pattern than in colour^37,69,70^. One potential reason for this could be that perception of colour depends on the visual system, and predators have different abilities (or constraints) in seeing wavelengths in natural light environments^60^. For example, some insects such as dragonflies, are visual hunters with markedly different visual abilities than birds, and that also prey on moths^71,72^. It is also known that insect predators use luminance contrast as a cue for their prey^73^. Thus, patterning might be a more universal target of selection as its detection is less sensitive to differences in colour perception of predators.

Most differences in pattern variability between aposematic and camouflaged species were also present among species with different flight times^5^. As most camouflaged moths were nocturnal, diel-activity and anti-predator colouration are often confounded. For example, camouflaged moths typically rest during daytime need effective camouflage when stationary^74,75^, and aposematic signals work more effectively under high light intensity^76,77^. As results between anti-predator colouration syndromes were generally more different than those between diel-activity, we focus our further discussion on predator selection on phenotypic variability of camouflaged and aposematic moths. Also, we did not find support for dietary niche influencing pattern variability for any traits.

While taking into account moth phylogeny, camouflaged moths showed more variable wing patterning than aposematic moths. The most variable pattern trait of camouflaged moths was marking size in both fore- and hindwings. Plausibly, pattern variability is important in defeating predator search image formation^22,23^ and could be linked to disruptive function^78–80^. More specifically, distant dependency may render colour pattern conspicuous at close viewing distances, but facilitate edge disruption and pattern blending into the background against low acuity visual predators by increasing distance^79,81,82^.Curiously, greater variability in hindwing patterning in camouflaged species went against our prediction that the wings that are most exposed (i.e., forewings) to diurnal predators should be more variable in camouflaged species. These results might point to weak or relaxed predator selection in nocturnal species on hindwing traits, which are rarely exposed to visual predators in camouflaged species during daytime. While we expressly removed from the data set species with camouflaged forewings and brightly coloured hindwings (startle defence), some nocturnal moths may have cryptic forewings with more uniform and brighter hindwing appearance, which might be used in signalling purposes^57^. Different functions of fore- and hindwings may thus partly explain the greater variability of hindwing patterning and fore- and hindwings may evolve independently^54–56^.

There are some provisos to our results. Efficient camouflage requires prey to match several visual characteristics of their habitat^29,30,83–85^, and for aposematic moths, the opposite is true; signals must include both internal (within animal contour) and external (animal-to-background) contrast^86,87^. As we lack data on visual backgrounds in this study, we could not directly test conspicuousness or background matching. Also, there are caveats about data from collection images. Although becoming more common, good-quality images are still scarce; for objective analysis museum photos should prioritise standard photographic conditions, uncompressed file-saving types, diffused natural light source, grey standards as well as scales. All of these were not always available (e.g., raw images are rarely available in large collections), although here we used only specimens that were in good condition and with no obvious fading or damage present (and a grey card and scale for image calibration). In addition, collections can be biased. Data portals sometimes include only one type of specimen per species, which is not ideal to measure phenotypic variation within and among species. In other words, some collections have oddities well-represented but may lack common phenotypes. Finally, for our categories, where data on the profitability of species were unavailable, we assigned varied green-toned (e.g., matching vegetation) or brown-toned (e.g., tree bark), species as camouflaged, and based aposematic category membership on knowledge of colouration role in closely related species, on opinion of experts in the group, or on generally supported syndromes of black and white with yellow or red as serving an aposematic role in moths^77,88^.

In summary, taking into account phylogenetic relationships, moth phenotypic intraspecific variability was best explained by anti-predator strategy, rather than diet breadth or even diel-activity. Our evidence supports the classic hypothesis^89,90^ that camouflaged moth species are more phenotypically variable than aposematic species, but much more so in pattern than colour. Several underlying mechanisms might explain these patterns in variability including frequency-dependent selection for and against phenotypic variation of camouflage and aposematism, respectively; determining which mechanism(s) underlie these differences are beyond the scope of this project. The activity patterns of moths and perceptual mechanisms of their predators add further layers of complexity on how the phenotypic variation gets filtered in the wild. The impressive range of colours and patterns in moths stem from selection via anti-predator defence and our work is the most thorough survey of the magnitude of intraspecific variability with this respect to date.

## Methods

To explore patterns in phenotypic variation of wing patterns in relation to anti-predator strategy, diel-activity patterns and diet breadth, we examined 2800 wing images of 82 moth species from eight moth families accessed via digitised online collections from natural history museums and universities through open-access biodiversity databases across the northern hemisphere (Supplementary Note 1). We use the term species for consistency although the species status of some of these moth taxa may not be clear. We also explore differences in mean values of hues, contrast and pattern in light of these ecological hypotheses, even though they are not part of the hypotheses we are trying to test, variance and means are often correlated, so examining mean values provides additional insights (Supplementary Note 2).

### Preconditions, selection and acquisition of samples

Included families were primarily selected based on the recognised aposematic or camouflaged status in literature as well as on sample size available in collections (Supplementary Data 1) resulting in 82 species from 8 moth families, 41 aposematic and 41 camouflaged species (2 factor levels). For categorisation, we sought experimental evidence on whether a species had been documented as having chemical defence, conspicuous appearance and/or was the subject of avoidance learning by predators (i.e., aposematism). Also, we searched to determine if a species had been shown to lack chemical defence or rely chiefly on background matching (i.e., camouflage). Information about the categorisation is found from the supplementary material (Supplementary Data 1–3). When species lacked these data, we searched for evidence on closely related species and consulted an expert who subjectively decided the category based on moths’ appearance guided by forewing coloration. We did not include species that may use startle in our sampling (e.g., we left *Catocala* moths that have cryptic forewings and conspicuous hindwings away). We used the families Crambidae, Erebidae, Geometridae, Lasiocampidae, Noctuidae, Sphingidae, Megalopygidae and Zygaenidae.

Regarding autecological attributes of these species, for diel-activity (3-factor levels), 18 species were cathemeral, 15 diurnal and 49 nocturnal. For larval dietary preference (4 factor levels), 12 species were monophagous herbivores (feed on a single genus of host), 14 oligophagous herbivores (feed on more than one genus in the same family) and 51 polyphagous herbivores (feed on more than one plant family) and 5 mycophagous (feed on fungi). Some moths complete their life cycle close to their host plants and thus, larval dietary niche may predict phenotypic associations in adults. We also justify using larval diets, because they may allow sequestration of toxic chemicals, which could be related to both aposematism and dietary specialisation, if sequestration requires adaptations to specific compounds (e.g., monarch butterflies). Less is known about moth adult feeding, but it tends to be more generalised as moths are often nectar feeders. With regard to ‘mycophagous’, these species could plausibly be specialists or generalists (Supplementary Data 3).

Sampling was done by utilising digitised collections of the Natural History Museum of London and the open-access biodiversity databases Global Biodiversity Information Facility (GBIF) and Symbiota Collections of Arthropods Network (SCAN) that facilitate access to digitised specimen collections of several museums and universities. The following collections were used: The Natural History Museum of London (49 species), Estonian Museum of Natural History (TAMZ— 14 species), Colorado State University, C. P. Gillette Museum of Arthropod Diversity (CSU_ENT—8 species), San Diego Natural History Museum (SDNHM—7 species), Arizona State University, Hasbrouck Insect Collection (ASUHIC—7 species), Yale University, Yale Peabody Museum (YPM ENT—4 species), Mississippi Entomological Museum (MEM—4 species), Michigan State University, The Albert J. Cook Arthropod Research Collection (MSUC_ARC—4 species), New Mexico State University, Collection of Arthropod (NMSUACP— 3 species), Northern Arizona University, The Colorado Plateau Museum of Arthropod Biodiversity (NAUF—2 species), Dugway Proving Ground Natural History Collection (DPG1HEXA—2 species), The Purdue Entomological Research Collection (PERC—1 species), University of California Santa Barbara Invertebrate Zoology Collection (UCSB-IZC—1 species), Academy of Natural Sciences Entomology Collection (ANSP-ENT—1 species), Entomology Collection at the Natural History Museum of Utah (UMNH.ent—1 species). The full list of sampling can be found from the supplementary information (Supplementary Data 2).

Phenotypic analyses on colour and pattern were performed with the ‘Multispectral Image Calibration and Analysis (MICA) Toolbox and Quantitative Colour and Pattern Analysis plugin^64^ in the open-source image processing programme ImageJ^65^. Digitised sample images were initially provided in JPG format. To ensure that image quality sufficed for image analysis, sample image sizes needed to be at least 1000 px in length and width, and on average image dimensions were 4700 × 3100 px. We focused on wings, because they were the most preserved part of specimens. The chosen moth specimens were in good condition; paying attention that wings were intact, colours had not faded, and wing scales were not scattered. Possible dimorphic sexes and colour morphs were included in the analysis to achieve a better understanding of the amount of variation in different phenotypes within each species. We aimed for at least 30 samples per morphotype (i.e., if moths differed in appearance by sex, subspecies or distinctive colour morphs). Some limitations in the availability of images forced us to downscale the sample size for some species. For example, female specimens appeared less abundant compared to male specimens—as they often are in museum collections^91^—and the goal of 20 samples was set for sampling of females and other groups for which supply was scarce. For four groups, sampling was between 14 and 18 images. Minimum wingspan of the selected moth species was decided to be at least 20 mm as for moths smaller than this measuring would have been less reliable from museum photographs. To summarise, the lowest number of samples per species was 14. Only 6% of all the species sampled (5/82 species) had fewer samples than 20 per species, 12% (10/82 species) had 20–30 samples, and 82% (67/82 species) had at least 30 samples (Supplementary Data 2).

Sampling of moth species took into account that species can often show geographic variation in phenotypes. Individuals representing a species were chosen from collections within a 500 km radius geographical proximity to one another (as sampling must include a decent range ecologically but must also minimise the influence of climate and other ecological differences –like changes in predator communities—on colouration), and that had digital images. The complete sample size of each species was usually taken from the same image provider museum collection (see above). The majority of the specimens we used were less than 90 years old. However, specimens held under controlled conditions maintain their appearance well in museum conditions, and only specimens that were in good condition and with no obvious fading or damage were used (as described earlier).

### Colour space and pattern analysis

Documentation of colour relies on information about light conditions, accuracy of reflectance recordings and sensitivity of vision to process colour. The choice of colour and pattern metrics and analysis therefore depend on the constraints of data and aims of the research.

We used images of specimens from entomological museum collections; we included only specimen images that had a calibration card (i.e., grey card and/or colour standard) that were taken with a DLSR digital camera that captures wavelengths in three channels (RGB). This methodology results in reflectance data spanning 400–700 nm and lacking UV; thus, we can only analyse patterns of intraspecific variability in colour across this range.

We note that different methodologies to analyse pattern variation exist. Some of them are more sensitive to detect orientation in pattern (e.g., stripes from spots), some deal with connectivity (i.e., adjacency analysis), some deal with edge disruption (e.g., GabRat filtering) and some provide parameters of granularity (e.g., marking size, contrast, dominance etc.). We chose to use granularity analysis using raw camera values (i.e., no vision model took place) on wing patterning and its variation to undertake a robust approach on certain key features. The limitations of this method are that it does not distinguish between different pattern types such as stripes and spots, but since pattern type is variable across moth species, the metric we have chosen can assess granularity (e.g., size differences) and contrast across a variety of patterns.

### Phenotypic data and image analysis

Since sample images were collected from different image providers, no accurate information could be gained from camera models and settings as is often the case^92^. Since images were taken using different cameras, they were all converted from JPG format to digital negative raw image (DNG) format with digital photography editing and management software Adobe® Lightroom®. Samples were photographed against a standard ‘middle grey’ photographic background and this middle grey (ca 40% reflectance) was used as grey standard for adjusting white balance in the sample images (i.e., to control for tonal range).

Processing and analysis of images were done with the programme ImageJ 1.53e utilising the image analysis tool micaToolbox Version v2.1.1^64,93^. Images were converted into multispectral image stacks with micaToolbox and regions of interest; the entire dorsal fore- and hindwing areas were selected. We conducted a multispectral image analysis on camera normalised responses to blue (B), green (G) and red (R) colour channels (i.e., short, medium and long wavelengths, respectively) and pattern variables (i.e., pattern dominance, pattern size, and pattern contrast) with micaToolbox Batch multispectral image analysis.

For colour analysis, the RGB channel values were converted to HSV colour space to better characterise colour space properties (i.e., hue, saturation and brightness, Supplementary Fig. 1). We caution that HSV is designed for computer vision use and operates in 400–700 nm range (i.e., human visible light, but it is not 1:1 human vision model). However, as a standard colour space, it helps us to characterise key differences in hue, saturation, and brightness, when accurate parameters for more sophisticated vision modelling are partially unknown. We cannot specify background matching or conspicuousness of moths in their natural visual environment with our data set, but nevertheless the chosen metrics robustly characterise phenotypic variation via different dimensions in colour space.

To extract pattern information, we applied a pattern (‘granularity’) analysis technique that decomposes an image into a series of spatial frequencies using Fourier analysis and band pass filtering and follows with determining the relative contribution of different marking sizes to the overall pattern^94–96^. Granularity analysis is a powerful tool for quantifying animal patterning as it objectively measures variation in phenotypic appearance in terms of arrangement in luminance composition^64,94–96^. The analysis was conducted by setting linear increments of one pixel from 1 to 100 pixels corresponding to the tenth of a millimetre increments up to 1 centimetre. This method controls for size variation and scales wings by a known pixel/millimetre ratio, which standardises size and thus yields isometric pattern values. We used 20 luminance bands range in pattern analyses. We used maxPower (i.e., dominance—the energy at the spatial frequency with the highest pixel energy), maxFreq (i.e., marking size—the spatial frequency with peak energy) and sumPower (i.e., contrast—the energy summed across all scales) of the micaToolbox pattern variables which here are considered as pattern dominance, marking size (or sizing) and contrast, respectively (Supplementary Fig. 1). Pattern analysis was conducted in custom files for ImageJ^64^.

### Phylogenetic topology

To take phylogenetic relationships into account in our statistical analyses, we generated an ultrametric topology of the sampled species. The constraint topology was generated manually based on published phylogenetic studies for each group, as well as for all Lepidoptera at the family level^97–101^. Noteworthily, the focus here is not the taxonomic status of these species, but to take into account their relatedness and branch lengths using ultrametric phylogenetic trees in the analysis. For branch length estimation, DNA sequences of up to eight gene fragments were downloaded from the NCBI database for each species. In a few cases, there were no sequences available for the sampled species, in which case we used sequences from a closely related species, ensuring that the phylogenetic structure of the samples species was preserved. We estimated branch lengths for the constraint topology using IQTREE, with the GTR + F model assigned to each gene partition separately. The topology with branch lengths was then made ultrametric using the R package *ape*^102^, with five nodes given age constraints based on ref. ^103^. We emphasise that this is not a formal timing of divergence analysis, but rather a way to get an ultrametric topology that approximates times of divergence of the sampled species. Given the highly skewed nature of taxon sampling and the amount of missing DNA sequence data, a formal analysis is not possible.

### Statistical analysis

All statistical analyses on the variability of colours and patterns of different species assemblages were performed using phylogenetic analysis of (co)variance, phylANOVA^104^, and implemented using the phytools package^105^ in the R programming language. This function performs a simulation-based phylogenetic ANOVA^104^ and compares groups controlled by an ultrametric phylogenetic tree (Fig. 2). As response variables, we calculated group means (x) and coefficient of variation (cv) for forewing and hindwing colour (hue, saturation, brightness) and pattern metrics (dominance, marking size, contrast) for each taxon from the camera-obtained image analysis data. We characterise phenotypic variability of moths through means and coefficient of variation and use these separately for forewings and hindwings in all analysis.

We tested whether any of the three ecological hypotheses: anti-predator function, diel-activity or dietary-niche, explained phenotypic variation of the moths. For this, we tested each of the phenotype metrics separately as response variable against the ecological explanatory variable: anti-predator function (aposematic, camouflaged), diel-activity (cathemeral, diurnal, nocturnal) or dietary-niche (monophagous, oligophagous, polyphagous, mycophagous) as predictor in the statistical phylANOVA model using sequential Bonferroni correction (“Holm-Bonferroni method)”. For example, the code used was as: phylANOVA(tree, x1, y1, nsim = 1000, posthoc = TRUE, p.adj = “holm”), where the phylANOVA is the phylogenetic ANOVA function, tree is the ultrametric phylogenetic tree, x1 is the predictor variable (or vector containing the groups) such as anti-predator function (or activity or diet), y1 is the response variable (or vector containing the response variable) such as forewing marking size coefficient of variation, nsim is an integer specifying the number of simulations, posthoc is a logical value whether or not to conduct posthoc tests to compare means among groups and p.adj is the method for the posthoc tests to account for multiple testing (we used sequential Bonferroni correction). The effect sizes were reported using Eta Squared (η2) test statistics.

### Reporting summary

Further information on research design is available in the Nature Portfolio Reporting Summary linked to this article.

### Supplementary information

Supplementary info

Peer Review File

Reporting Summary

Description of Additional Supplementary Files

Supplementary Data 1

Supplementary Data 2

Supplementary Data 3

### Source data

Source Data

## Data Availability

All data are available in Jyväskylä University Digital Repository JYX (10.17011/jyx/dataset/92453). Source data are provided with this paper.
