# Supplementary material for: Predator selection on phenotypic variability of cryptic and aposematic moths
Source: Nat Commun. 2024 Feb 23;15:1678. doi: 10.1038/s41467-024-45329-5 (PMC10891176; doi:10.1038/s41467-024-45329-5)
Supplement: Supplementary file 1 — Supplementary info [file 41467_2024_45329_MOESM1_ESM.pdf]

Supplementary information for

Predator selection on phenotypic variability of cryptic and aposematic moths

Ossi Nokelainen[1,2,3*], Sanni A. Silvasti[2,4*], Sharon Y. Strauss[5,6], Niklas Wahlberg[7] & Johanna Mappes[1,6*]

[1]University of Helsinki, Organismal and Evolutionary Biology Research Programme, Faculty of Biological and Environmental Sciences, Viikki Biocenter 3, P.O. Box 65, 00014 University of Helsinki, Helsinki, Finland

[2]University of Jyväskylä, Dept. of Biological and Environmental Science, P.O. Box 35, FI-40014 Jyväskylä, Finland

[3]University of Jyväskylä, Open Science Centre, P.O. Box 35, FI-40014 Jyväskylä, Finland

[4]Macquarie University, Dept. of Biological Sciences, Sydney, New South Wales 2109, Australia

[5]University of California at Davis, Department of Evolution and Ecology, 2320 Storer Hall, One Shields Avenue, Davis, CA 95616, USA

[6]Wissenschaftskolleg zu Berlin, Wallotstrasse 19, Berlin, Germany 14193

[7]Lund University, Dept. of Biology, Sölvegatan 37, SE-223 62 Lund, Sweden

Copyright: All authors listed and affiliated universities. Published by Nature Portfolio.

Licence: Creative Commons Attribution 4.0 International (CC-BY 4.0)

Supplementary Information Contents: Page

Fig. 1: A theoretical depiction of the key parameters measured 2

Fig. 2: The significant phenotype metrics and their group means 3

Fig. 3: Colour pattern variation of the phenotypic differences 4

Fig. 4: A heatmap plot of the significant associations 5

Note 1: List of image collection used in the sampling 6

Note 2: The differences in colour and pattern mean values 7

Supplementary Fig. 1: A theoretical depiction of the key parameters measured. A) The first panel shows the conical HSV-colour space whereby colour is characterised by hue (dominant wavelenght), saturation (colour purity) and the value (brightness). B) The second panel simplifies the pattern (or 'granularity') analysis, whereby pattern is characterised by marking size (i: max Freq - the spatial frequency with the highest energy; i.e., corresponding to the dominant marking size), pattern dominance (ii: maxPower - the pixel energy at maximum frequency) and pattern contrast (iii: sumPower - the energy summed across all scales, a measure of pattern contrast).

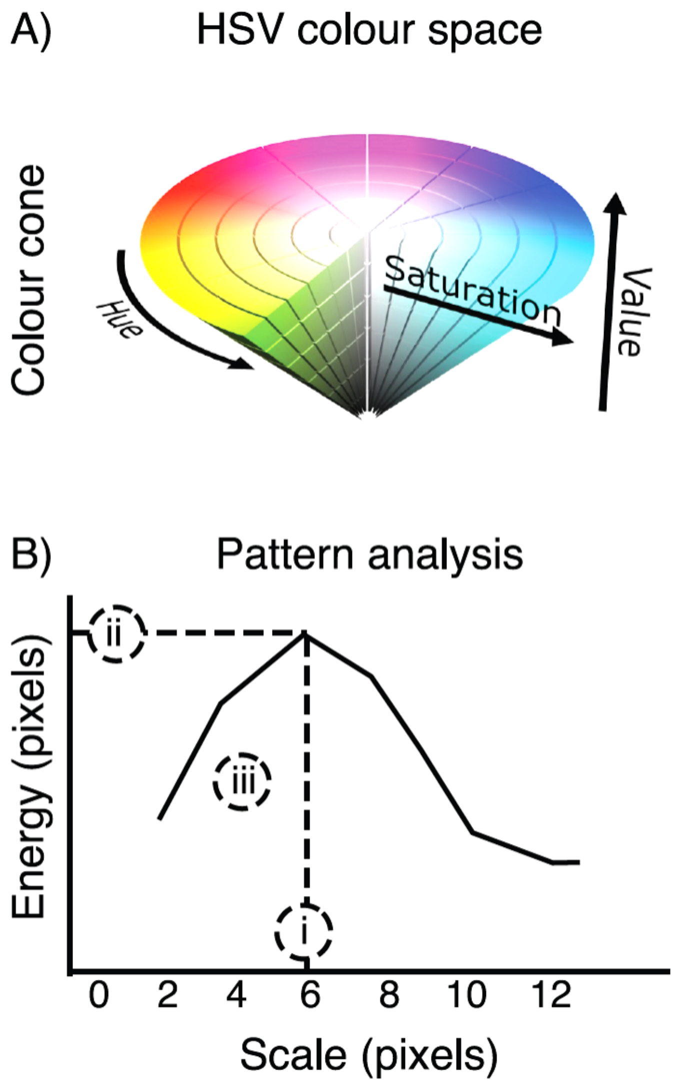

Supplementary Fig. 2: The significant phenotype metrics and their group means (x). Wing pattern (or marking) size values are shown for fore- and hindwings. Boxplots are organised in descending order with respect to test statistics and separate anti-predator strategies and moth diel-activity. The boxplot shows minimum and maximum (whiskers), median line and the interquartile range of the data.

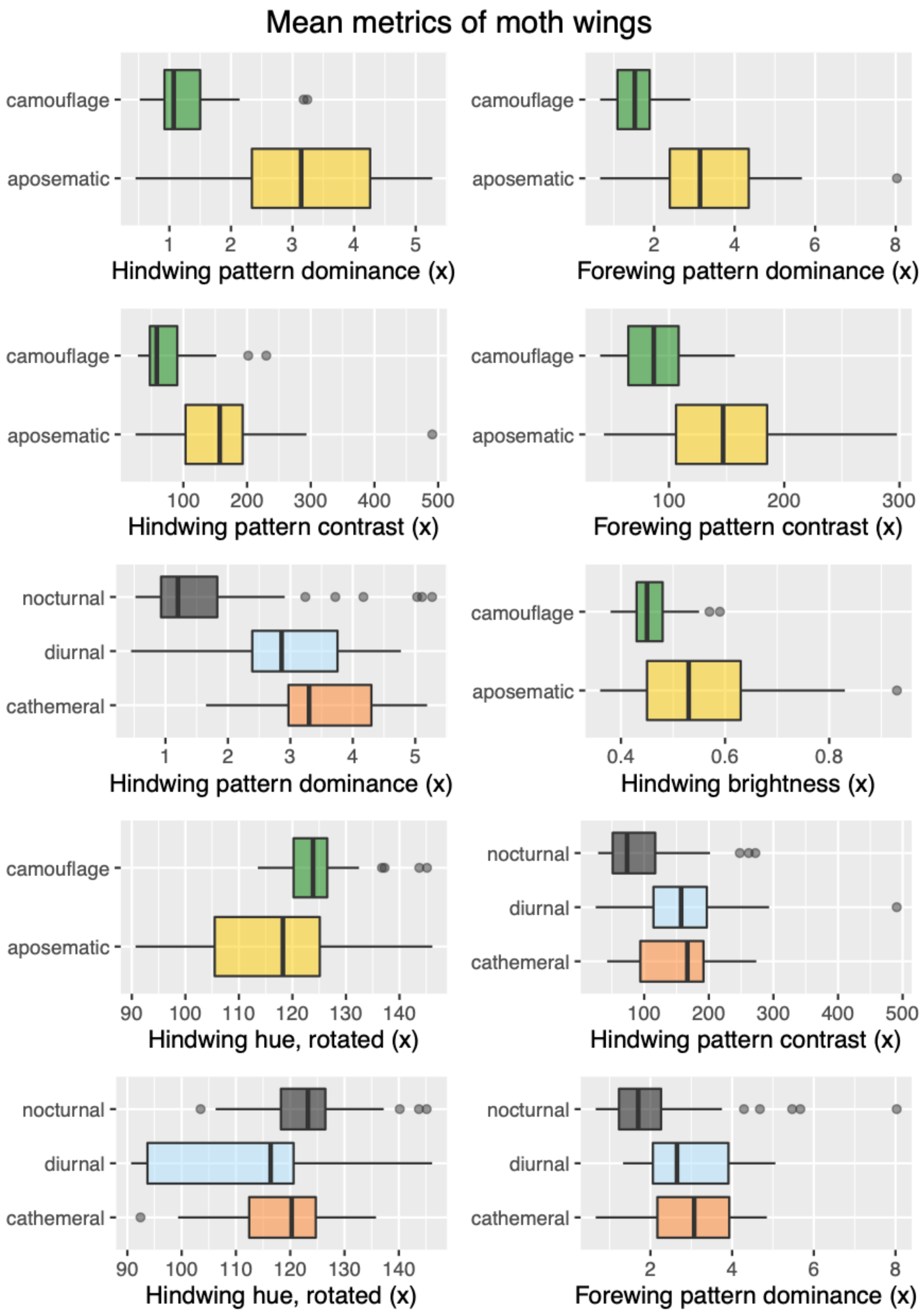

Supplementary Fig. 3: Camera-obtained data summarising moth colour and pattern variation of the phenotypic differences. Values are separated for fore (FW) and hind wing (HW) regions of interest (ROIs). The data is characterised through mean (x) for interspecific variation and coefficient of variation (cv) for intraspecific variability. The class separates aposematic (apo) and camouflage (cam) strategies and moth diel activity: nocturnal (noc), cathemeral (cat) and diurnal (diu).

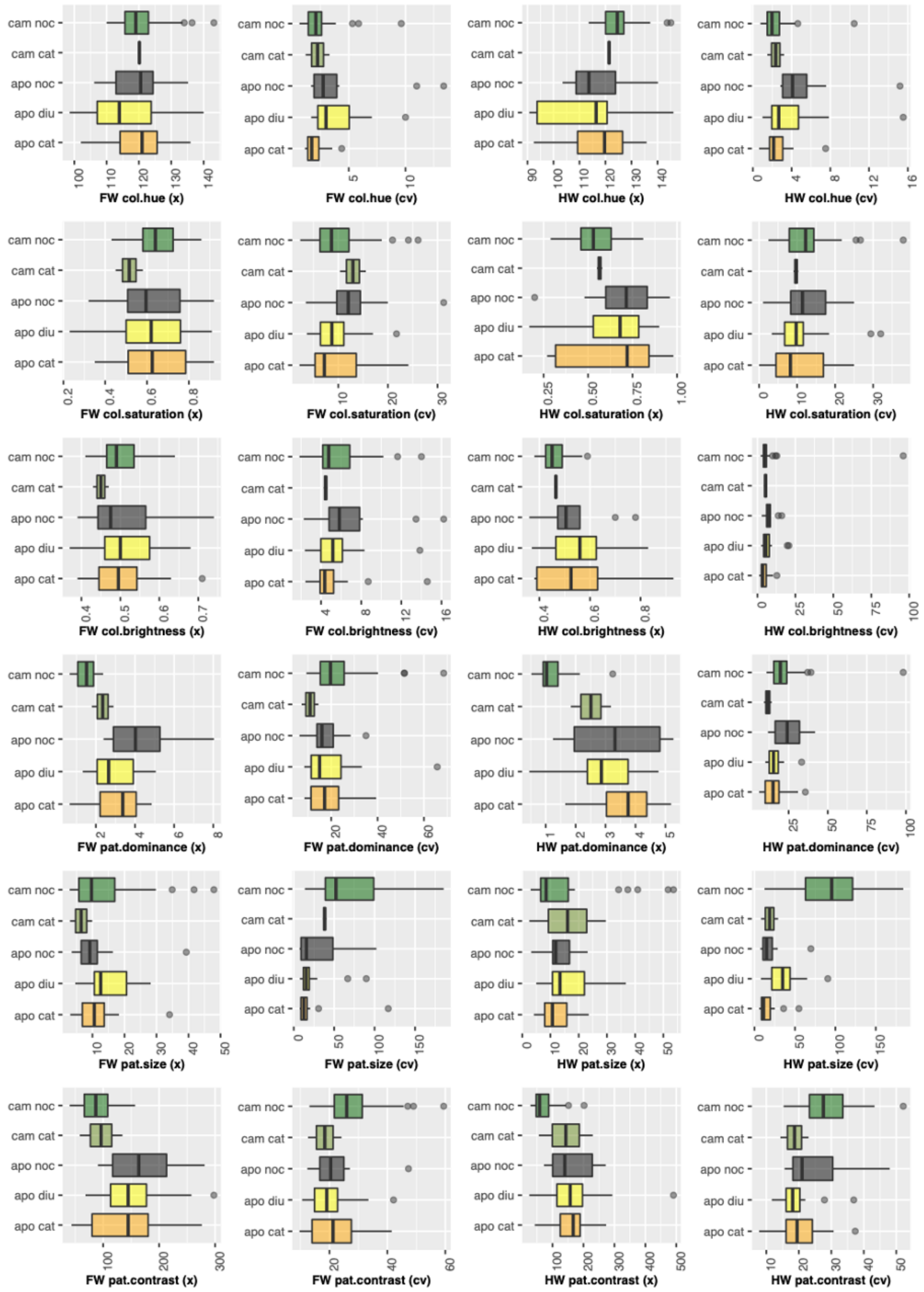

Supplementary Fig. 4: A heatmap plot for significant associations of the moth colour and pattern as regards of the three ecological hypotheses predicting their appearance. On the x-axis values stands for forewing and hindwing, x for mean (i.e., interspecific variation) and cv for coefficient of variation (i.e., intraspecific variation). On the y-axis, different pattern and colour metrics are compared against their alternative hypotheses for moth phenotypic variability: anti-predator strategy, diel-activity and dietary-niche. The panel shows F-statistics and significant p-values from the phyloANOVA analysis (i.e., the higher F-values and lower p-values indicate higher statistical significance).

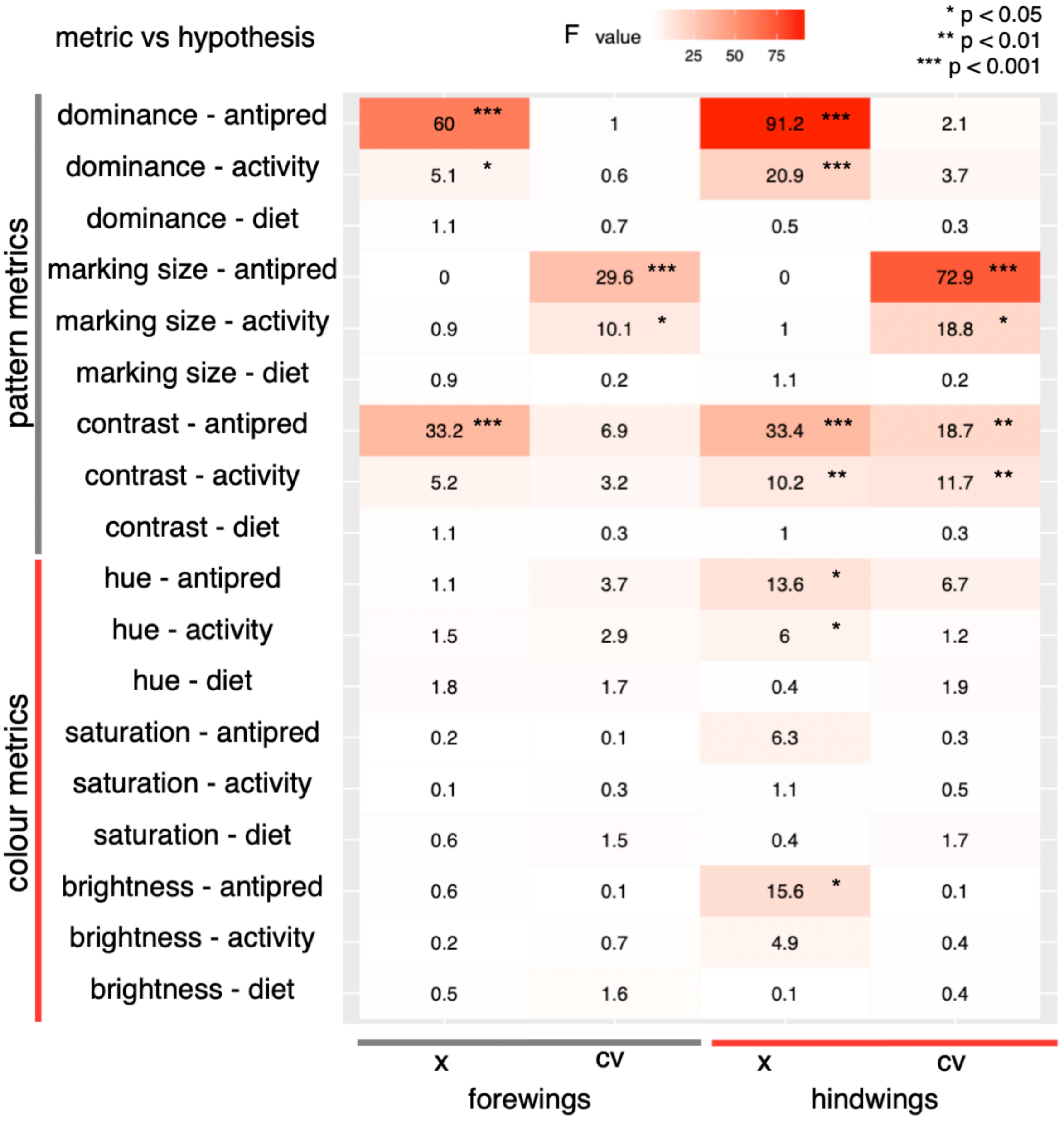

Supplementary Note 1: Variability of moths - List of image collection used in the sampling.

The Natural History Museum of London
- 49 species

Estonian Museum of Natural History (TAMZ)
- 14 species

Colorado State University, C. P. Gillette Museum of Arthropod Diversity (CSU_ENT)
- 8 species

San Diego Natural History Museum (SDNHM)
- 7 species

Arizona State University, Hasbrouck Insect Collection (ASUHIC)
- 7 species

Yale University, Yale Peabody Museum (YPM ENT)
- 4 species

Mississippi Entomological Museum (MEM)
- 4 species

Michigan State University, The Albert J. Cook Arthropod Research Collection (MSUC_ARC)
- 4 species

New Mexico State University, Collection of Arthropod (NMSUACP)
- 3 species

Northern Arizona University, The Colorado Plateau Museum of Arthropod Biodiversity (NAUF)
- 2 species

Dugway Proving Ground Natural History Collection (DPG1HEXA)
- 2 species

The Purdue Entomological Research Collection (PERC)
- 1 species

University of California Santa Barbara Invertebrate Zoology Collection (UCSB-IZC)
- 1 species

Academy of Natural Sciences Entomology Collection (ANSP-ENT)
- 1 species

Entomology Collection at the Natural History Museum of Utah (UMNH.ent)
- 1 species

Supplementary Note 2: On the interspecific differences in mean values of colour and pattern

In pattern trait means, aposematic moths had more dominant patterning (Strategy - Dominance.x. Forewing: $F = 59.97$, $\eta2 = 0.42$, $p = 0.001$; hindwing: $F = 91.19$, $\eta2 = 0.53$, $p = 0.001$) and pattern contrast than camouflaged species (Strategy - Contrast.x. Forewing: $F = 33.21$, $\eta2 = 0.29$, $p = 0.001$; hindwing: $F = 33.38$, $\eta2 = 0.29$, $p = 0.001$). Nocturnal and cathemeral lineages also had greater pattern dominance than diurnal species (Activity - Dominance.x. Forewing: $F = 5.07$, $\eta2 = 0.11$, $p = 0.073$; hindwing: $F = 20.93$, $\eta2 = 0.34$, $p = 0.001$). Nocturnal camouflaged moths had least contrasting hind wing patterns (Activity - Contrast.x. Forewing: $F = 5.17$, $\eta2 = 0.11$, $p = 0.066$; hindwing: $F = 10.18$, $\eta2 = 0.20$, $p = 0.008$, Figure 5). There were no significant differences in mean values of marking size (Strategy – Marking size.x. Forewing: $F = 0.01$, $\eta2 < 0.01$, $p = 0.979$; hindwing: $F = 0.01$, $\eta2 < 0.01$, $p = 0.958$, Activity – Marking size.x. Forewing: $F = 0.93$, $\eta2 = 0.02$, $p = 0.624$; hindwing: $F = 0.99$, $\eta2 = 0.02$, $p = 0.609$).

In colour traits, the key differences were in the hindwing colour: aposematic moths had significantly different hindwing colour hue (i.e., the dominant wavelength) than camouflaged species (Strategy - Hue.x. Forewing: $F = 1.13$, $\eta2 = 0.01$, $p = 0.513$; hindwing: $F = 13.62$, $\eta2 = 0.14$, $p = 0.018$). Also, mean hue values varied more among aposematic moths and especially in diurnal lineages (Activity - Hue.x. Forewing: $F = 1.48$, $\eta2 = 0.03$, $p = 0.448$; hindwing: $F = 5.97$, $\eta2 = 0.13$, $p = 0.045$). Mean brightness of hindwings was significantly higher in aposematic over camouflaged moths (Strategy - Brightness.x. Forewing: $F = 0.57$, $\eta2 = 0.01$, $p = 0.629$; hindwing: $F = 15.61$, $\eta2 = 0.16$, $p = 0.011$, Activity - Brightness.x. Forewing: $F = 0.17$, $\eta2 < 0.01$, $p = 0.929$; hindwing: $F = 4.85$, $\eta2 = 0.10$, $p = 0.077$). In terms of mean saturation, there was no statistically significant differences (Strategy – Saturation.x. Forewing: $F = 0.15$, $\eta2 < 0.01$, $p = 0.775$; hindwing: $F = 6.26$, $\eta2 = 0.07$, $p = 0.136$, Activity – Saturation.x. Forewing: $F = 0.13$, $\eta2 < 0.01$, $p = 0.943$; hindwing: $F = 1.12$, $\eta2 = 0.02$, $p = 0.565$).
