# Supplementary material for: Predator selection on phenotypic variability of cryptic and aposematic moths
Source: Nat Commun. 2024 Feb 23;15:1678. doi: 10.1038/s41467-024-45329-5 (PMC10891176; doi:10.1038/s41467-024-45329-5)
Supplement: Supplementary file 3 — Reporting Summary [file 41467_2024_45329_MOESM3_ESM.pdf]

nature portfolio

Corresponding author(s): Ossi Nokelainen & Johanna Mappes

Last updated by author(s): 15.1.2024

Reporting Summary

Nature Portfolio wishes to improve the reproducibility of the work that we publish. This form provides structure for consistency and transparency in reporting. For further information on Nature Portfolio policies, see our Editorial Policies and the Editorial Policy Checklist.

Statistics

For all statistical analyses, confirm that the following items are present in the figure legend, table legend, main text, or Methods section.

n/a	Confirmed	
☐	☒	The exact sample size (*n*) for each experimental group/condition, given as a discrete number and unit of measurement
☐	☒	A statement on whether measurements were taken from distinct samples or whether the same sample was measured repeatedly
☐	☒	The statistical test(s) used AND whether they are one- or two-sided<br>*Only common tests should be described solely by name; describe more complex techniques in the Methods section.*
☐	☒	A description of all covariates tested
☐	☒	A description of any assumptions or corrections, such as tests of normality and adjustment for multiple comparisons
☐	☒	A full description of the statistical parameters including central tendency (e.g. means) or other basic estimates (e.g. regression coefficient) AND variation (e.g. standard deviation) or associated estimates of uncertainty (e.g. confidence intervals)
☐	☒	For null hypothesis testing, the test statistic (e.g. *F*, *t*, *r*) with confidence intervals, effect sizes, degrees of freedom and *P* value noted<br>*Give P values as exact values whenever suitable.*
☒	☐	For Bayesian analysis, information on the choice of priors and Markov chain Monte Carlo settings
☐	☒	For hierarchical and complex designs, identification of the appropriate level for tests and full reporting of outcomes
☐	☒	Estimates of effect sizes (e.g. Cohen's *d*, Pearson's *r*), indicating how they were calculated

Our web collection on statistics for biologists contains articles on many of the points above.

Software and code

Policy information about availability of computer code

Data collection	Quantitative Colour & Pattern Analysis: MicaToolbox plugin (2.1.1) in ImageJ (1.53e, image analysis), Adobe Lightroom (image pre-processing).
Data analysis	R packages: ape, phytools, ggplot2, ggpubr, ggtree. All data was analysed with program R (version 4.2.1, R Core Team 2022)

For manuscripts utilizing custom algorithms or software that are central to the research but not yet described in published literature, software must be made available to editors and reviewers. We strongly encourage code deposition in a community repository (e.g. GitHub). See the Nature Portfolio guidelines for submitting code & software for further information.

Data

Policy information about availability of data

All manuscripts must include a data availability statement. This statement should provide the following information, where applicable:

- Accession codes, unique identifiers, or web links for publicly available datasets
- A description of any restrictions on data availability
- For clinical datasets or third party data, please ensure that the statement adheres to our policy

Data is available at University of Jyväskylä data repository (10.17011/jyx/dataset/92453).

Research involving human participants, their data, or biological material

Policy information about studies with human participants or human data. See also policy information about sex, gender (identity/presentation), and sexual orientation and race, ethnicity and racism.

Reporting on sex and gender	n/a
Reporting on race, ethnicity, or other socially relevant groupings	n/a
Population characteristics	n/a
Recruitment	n/a
Ethics oversight	n/a

Note that full information on the approval of the study protocol must also be provided in the manuscript.

Field-specific reporting

Please select the one below that is the best fit for your research. If you are not sure, read the appropriate sections before making your selection.

☐ Life sciences ☐ Behavioural & social sciences ☒ Ecological, evolutionary & environmental sciences

For a reference copy of the document with all sections, see nature.com/documents/nr-reporting-summary-flat.pdf

Ecological, evolutionary & environmental sciences study design

All studies must disclose on these points even when the disclosure is negative.

Study description	The work involves investigating moth wing images originating from three online museum collections. We asked whether anti-predator strategy (aposematism, camouflage), dietary specialization (monophagous, mycophagous, oligophagous, polyphagous) or flight activity patterns (diurnal, cathemeral or nocturnal) could explain differences in phenotypic variability of 82 Holarctic moth species while controlling for phylogeny. In this work, taking into account phylogenetic relationships, moth phenotypic variability is best explained by anti-predator strategy with camouflaged moths being more variable in wing patterning than aposematic species.
Research sample	We opportunistically exploited entomological online collections. Digital specimens that had intact wings and were in good condition were obtained from museum databases. Each species was collected within a 500 km proximity to control geographic variation.
Sampling strategy	Digital images of approximately 2800 moth wings from 82 species obtained from online museum collections were used to provide information about moth inter- and intraspecific phenotypic variation. No specific power analysis was conducted to address the ecological questions prior to analysis. We aimed for 41 aposematic and camouflaged moths and approximately 30 samples per species. Samples were opportunistically collected while visually evaluating their condition from images. Minimum wingspan of the selected moth species was decided to be at least 20 mm. The further details are described in the methods.
Data collection	Images were obtained from entomological online collections by Sanni Silvasti and image analysis was performed by Ossi Nokelainen.
Timing and spatial scale	Less than 90 years old samples from Holarctic distribution.
Data exclusions	No wing data were excluded from analysis.
Reproducibility	To summarize, we aimed for at least 30 samples per species; the lowest number of samples per species was 14. Only 6 percent of all the species sampled (5/82 species) had fewer samples than 20 per species, 12 percent (10/82 species) had 20-30 samples, and 82 per cent (67/82 species) had at least 30 samples. The sample sizes are listed in supplementary materials (Supplementary data 2).
Randomization	Image acqusition was randomised.
Blinding	Different person conducted analyses than who pre-processed images.

Did the study involve field work? ☐ Yes ☒ No

Reporting for specific materials, systems and methods

We require information from authors about some types of materials, experimental systems and methods used in many studies. Here, indicate whether each material, system or method listed is relevant to your study. If you are not sure if a list item applies to your research, read the appropriate section before selecting a response.

Materials & experimental systems

n/a	Involved in the study	
x		Antibodies
x		Eukaryotic cell lines
x		Palaeontology and archaeology
	x	Animals and other organisms
x		Clinical data
x		Dual use research of concern
x		Plants

Methods

n/a	Involved in the study	
x		ChIP-seq
x		Flow cytometry
x		MRI-based neuroimaging

Animals and other research organisms

Policy information about studies involving animals; ARRIVE guidelines recommended for reporting animal research, and Sex and Gender in Research

Laboratory animals	n/a
Wild animals	n/a: entomological museum collections
Reporting on sex	Findings do not discriminate sex.
Field-collected samples	n/a: entomological museum collections
Ethics oversight	No ethical approval required for online museum collection use.

Note that full information on the approval of the study protocol must also be provided in the manuscript.

Plants

Seed stocks	n/a
Novel plant genotypes	n/a
Authentication	n/a
