# Supplementary material for: Predator selection on phenotypic variability of cryptic and aposematic moths
Source: Nat Commun. 2024 Feb 23;15:1678. doi: 10.1038/s41467-024-45329-5 (PMC10891176; doi:10.1038/s41467-024-45329-5)
Supplement: Supplementary file 4 — Description of Additional Supplementary Files [file 41467_2024_45329_MOESM4_ESM.pdf]

Description of Additional Supplementary Files

File Name: Supplementary Data 1
Description: Autecological attributes of the moth species. Forty-one species were aposematic and 41 were camouflaged. For diel-activity, 18 species were cathemeral, 15 diurnal and 49 nocturnal (3 factor levels). For dietary-preference, 12 species were monophagous, 5 mycophagous, 14 oligophagous and 51 polyphagous (4 factor levels).

File Name: Supplementary Data 2
Description: Aposematic and camouflaged moth species: country and database of origin, their wingspans, and literature on defence mechanisms.

File Name: Supplementary Data 3
Description: Notes and descriptions of species according to their food plant and activity patterns: species, activity, diet breadth, plant growth form, plant family, plant genera, diet reference, diet notes, activity reference and activity notes.
