# Supplementary material for: Predator selection on phenotypic variability of cryptic and aposematic moths
Source: Nat Commun. 2024 Feb 23;15:1678. doi: 10.1038/s41467-024-45329-5 (PMC10891176; doi:10.1038/s41467-024-45329-5)
Supplement: Supplementary file 2 — Peer Review File [file 41467_2024_45329_MOESM2_ESM.pdf]

nature portfolio

Peer Review File

Predator selection on phenotypic variability of cryptic and aposematic moths

Open Access This file is licensed under a Creative Commons Attribution 4.0 International License, which permits use, sharing, adaptation, distribution and reproduction in any medium or format, as long as you give appropriate credit to the original author(s) and the source, provide a link to the Creative Commons license, and indicate if changes were made. In the cases where the authors are anonymous, such as is the case for the reports of anonymous peer reviewers, author attribution should be to 'Anonymous Referee' followed by a clear attribution to the source work. The images or other third party material in this file are included in the article's Creative Commons license, unless indicated otherwise in a credit line to the material. If material is not included in the article's Creative Commons license and your intended use is not permitted by statutory regulation or exceeds the permitted use, you will need to obtain permission directly from the copyright holder. To view a copy of this license, visit http://creativecommons.org/licenses/by/4.0/.

REVIEWER COMMENTS

Reviewer #1 (Remarks to the Author):

I enjoyed reading this manuscript. The authors take an important question about adaptive evolution and put it to test with a rigorous and imaginative approach.

Hypotheses are well-formulated, and make sense. I did, however, wonder why size was not included as an explanatory variable; larger objects could be easier to detect & identify. I don't think it is necessary to include that here, but worth consideration.

I am happy with methodologies

Sometimes the writing could do (in my view) with some amendment. (do see the document with annoations submitted).

Reviewer #2 (Remarks to the Author):

In this paper, the author(s) present a detailed analysis of intraspecific colour pattern variation in the forewings and hindwings of 41 aposematic (conspicuous, presumed defended) and 41 camouflaged (cryptic, presumed undefended) species drawn from 8 Lepidoptera families. The sampled species were further classified in terms of their diel activity (cathemeral, diurnal or nocturnal) and their diet (monophagous, mycophagous, oligophagous or polyphagous). There were three a priori hypotheses proposed that might explain differences in the magnitude of intraspecific variation between species, based on (a) the nature frequency-dependent selection given the anti-predator strategy (aposematism should favour low variation, camouflage should favour high variation), (b) a diel activity:anti-predator defence interaction caused by the signalling environment (the author(s) proposed that there may be a difference in phenotypic variation between diurnal and nocturnal species, but only in aposematic moths) and (c) the nature of the heterogeneous selection experienced (a single host plant should favour less variation). The author(s) also investigated interspecific variation in phenotype between species, anticipating that aposematic species would tend to have greater mean contrast than camouflaged species once one controls for phylogeny (the results are summarized in the SI). Taking into account phylogenetic relationships, the author(s) proposed that the differences in intraspecific phenotypic variability observed (notably, aspects of pattern) were associated with differences in anti-predator strategy, and also diel activity (which is associated with anti-predator strategy) but not dietary niche.

The author(s) are to be congratulated for taking on such an ambitious project. Overall, the work is well thought through, clearly presented and the results are believable. Most of the (understandable) weaknesses of the study (e.g. lack of UV component, uncertainty over the natural backgrounds of the moth species) have already been highlighted and discussed, and the author(s) have taken a deep dive into what is known about each moth species. I particularly appreciate keeping observations of a given species to within a 500 km radius given the regional variation observed in many aposematic species (e.g. certain burnet moth species). The fact that both forewings and hindwings were considered is also to the author(s) credit. However, I feel that there are some aspects of the paper that could do with improvement. Below I make some comments and pose some questions which I hope will be of use to the author(s) in making an even clearer and better argued paper.

Statistics
The coefficient of variation works well as a standardized measure of dispersion, but it is a biased measure of the equivalent measure within a population (see Sokal & Rohlf 2012 Biometry 4th Edition page 56). The bias is small for large samples, but “appreciable” for small samples. Specifically, when a variable is normally distributed at population level, a correction factor of (1 + 1/4n) to the sample CV

provides the ML estimate of the population-level CV. If all species had the same sample size, then it would be a fixed adjustment of no consequence (all species equally underestimated to the same small degree when n is large). However, since n varies from 30 to 14, it might marginally under-estimate the population-level CV more in the less well represented species compared to better-represented species. So long as certain classes of moth were not especially under-represented it is unlikely to change anything, but perhaps worth bearing in mind. Moreover, as Sokal & Rohlf (2012) and multiple sources stress, the CV only has meaning for variables measured on the ratio scale i.e. where there is a true zero and the zero refers to the absence. This seems to be the case for the HSV (where 0,0,0 = black) and it would be worth confirming the 0's refer to absences in all the other more complex measures such as those based on granularity.

Since (a) the three hypotheses are not mutually exclusive (line 13), (b) the predictors were co-linear (aposematic species are more likely to be diurnal, line 189) and (c) one of the stated predictions was based on a diel activity: anti-predator strategy interaction (line 80), I wonder why models were separately fitted with single predictors (strategy, activity, diet) rather than fitting a full model with all predictors, including interactions that have been recognized in advance. That way, we reduce the number of models fitted, we can see whether diel activity explains the CV of species over and above their anti-predator strategy and we can test directly for the interaction that was anticipated. Of course, if diel activity and anti-predator strategy are highly co-linear the predictors may obscure one another's effects, but this would not be a problem if the variance could be sequentially partitioned and the predictors were entered in different orders.

The red highlighting of high F values in Figure 4 helps draw the eye to big differences between factor levels compared to variation within factor levels but note that the variance ratio is only a crude metric of effect size (the Eta-squared statistic provides a more principled measure). Of course, since the numerator degrees of freedom (and possibly the denominator df) differ for the NHST tests being displayed, the same value F could be considered consistent with the NH for tests involving 2 classes (1 df e.g. anti-predator strategy) than but not for tests involving 4 classes (3 df e.g. diet), say.

I assume that the MS that is widely reported in the results refers to the "mean square", but it is not very meaningful in itself (what does it mean if the MS is 40584.73 or 40.5?). It would be better to give an indication of the average effect size and provide the two degrees of freedom associated with the F-ratio.

Methods

It is a little unclear to me how the classifications of each moth species were arrived at. In particular, species are listed as aposematic or cryptic in the SI, although the associated summaries often state "No literature on aposematism" or "No literature on camouflage". How were these classifications made if there was no literature to go on? I may have missed this. If the classification was based on judgement of conspicuousness, who judged it and how? Looking at the images it may have been a clear-cut distinction, but I've seen enough moths to know that it isn't always the case especially given uncertainty over backgrounds. In addition, since some moth species have highly conspicuous hindwings used to startle would-be predators (line 205), I'm guessing that if a personal classification was adopted it was based on forewing colour patterns.

We see moth species listed as monophagous, mycophagous, oligophagous or polyphagous. However, adult moths (which have probiscis) often feed (if at all) in different places and on very different resources (nectar, sap, fruit) than their larvae (which have mandibles). It seems to me that in the context of the hypotheses proposed to explain wing pattern variation, the diet that matters is that of the adult not the larvae. Unfortunately I cannot find any mention of the life stage to which the diet refers. Naturally some moths complete their life cycle close to their host plant so arguably larval diet can say something about adult plant associations - but I don't see any arguments of this nature in the

text. Arguably, it could be a reason why the authors don't see a strong diet specialization effect! The classification "mycophagous" is also an odd one as it doesn't refer to diet breadth per se (can you be a mycophagous specialist or generalist?); a sentence to justify it would therefore help. Finally, larval monophagy is often difficult to pin down – indeed, several of the species listed a monophagous in Table S2 have been found on closely related host plant species. In short, more details are needed as to how the classifications were arrived at.

Minor details

Line 23 (and elsewhere) is it a truly a "hypothesis" that camouflaged moth species are more phenotypically variable than aposematic species or a prediction? Is it not a prediction that arises from a broader belief? Surely the hypothesis is that predatory behaviour to deal with an anti-predator strategy generates a form of frequency-dependence which either promotes diversity (search image) or selects against it (avoidance learning)? If true, it makes a certain predictions (which can be tested) regarding the degree of intra-specific variation in species that employ different anti-predator strategies strategies.

Line 66 AT the same time..

Line 66 Arguably the regional polymorphism does "fit ..the classic paradigm" since it is entirely consistent with positive frequency selection (strength in numbers).

I hope this feedback is of use.

Following Nat Comms stated preference, I am signing my review.

Tom Sherratt

Reviewer #3 (Remarks to the Author):

In this MS, the authors examined how antipredator strategy, activity, and diet influence intraspecific pattern and color variability among Lepidopterans. They found that antipredator strategy was the strongest predictor of pattern variability (aposematic species were less variable than camouflaged species), nocturnal species were more variable than cathemeral or diurnal species, and there were no effects of any covariate on color variation. Overall I found the paper interesting and the results mostly intuitive. My general criticisms on the paper are that there are many instances where the authors need to provide more clarity or exposition as it often reads as though it were written for a more specialist journal where readers have strong background knowledge of Lepidopterans and the deep literature on the evolution of animal coloration (not a Nature journal). Second, there were obvious grammatical issues throughout the manuscript so the paper needs a very thorough revision to correct these errors.

Comments:
L2: The start of the abstract is too abrupt/specific - it should be more general about coloration evolution and aposematic and camouflage strategies.

L37: Citations needed to support this sentence.

L50-53: I'm not sure I would call this relaxed selection. Relaxed selection implies that there was once strong selection for something and then that lessened. What the authors describe here is that there is simply general selection for particular colors and pattern elements, but weak overall selection (i.e., not stabilizing) governing how those colors/pattern elements come together.

L79-81: Explain why - this isn't intuitive to me.

Results: Since the F values are in the Figure, they aren't required to report in the results. It's also not evident what MS is. This section can be shortened if more of these stat results are put into the figure.

L267: please describe the standardized way that images were taken - list all of the criteria used.

L302: I don't understand why images for a species were all selected from a single geographic location. If you want to examine species variability, wouldn't you want to gather images of that species from across its range to indicate intraspecific variation??

L318-323: Please define these more clearly, especially granularity. It is not something that's known to people that don't specialize in coloration. What is dominance??? The granularity parameters need to be clearly defined and listed before they are discussed in the results.

Figure 4: Change "strategy" to antipred or ap-strategy

(NCOMMS-23-16093)

*****Reviewer 1 Remarks to the Author

I enjoyed reading this manuscript. The authors take an important question about adaptive evolution and put it to test with a rigorous and imaginative approach. Hypotheses are well-formulated, and make sense. I did, however, wonder why size was not included as an explanatory variable; larger objects could be easier to detect & identify. I don't think it is necessary to include that here, but worth consideration. I am happy with methodologies. Sometimes the writing could do (in my view) with some amendment. (do see the document with annoations submitted)

Our response: We are happy to see that the reviewer liked our paper, and we thank him/her for constructive comments. We find it plausible that when the moth size grows this is expected to lead into different requirements for achieving camouflage or warning signal. In fact, we did look on the size of specimens as independent variable, but as its role was not significant in our dataset (E.g., p-values ranging from 0.231 to 0.93) and the parameter not being crucial to our key focus, it was discarded from further analysis. However, we have now added this result to the paper (under "Differences in within-species variability") as: "Noteworthily, moth size measured as wing area was not significantly explained by any of the three ecological predictors." (Lines 134-136).

In addition, pattern variables were normalised into isometric values taking size into account through px/mm-size ratio. Our design was aimed at comparing species that are considered chiefly aposematic or camouflaged while considering their phenotypic variability, temporal activity, dietary preference, and phylogenetic background. We have also added more details about size as follows (Lines 373-375): "This method controls for the size variation and scales the wings with known pixel / millimetre ratio, which standardises the specimen's size and thus yields isometric pattern values".

1) I wonder if you could add in as follows - "negative frequency-dependent variation, in which rarity of appearance favours survival from predation" Also - was Poulton writing about camouflage or about all colourations? Here & later, the term Search Image does come with some cognitive-psychology baggage and some contention, and I wonder whether you should find a less loaded term - visual familiarity? Also, should you define aposematism here? Many interested readers may not know what it is, or be vague.

Our response: Unfortunately, we had to omit the first seven lines of the initial abstract due to abstract word count limitation. We have written the opening lines of the abstract now as: "Natural selection generally favours phenotypic variability in camouflaged organisms, whereas aposematic organisms are expected to evolve a more uniform warning coloration. However, no comprehensive analysis of the phenotypic consequences of predator selection in aposematic and cryptic species exists.".

2) So SI refers to very specialsied and transient cognitive/sensory specialisation; I doubt if +ve fds needs SI's specifically; rather memory for prey types

Our response: We have specified the sentence as regards to predators and memory for prey types as follows (Lines 39-41): "Search images (as memory for prey types) are

thought to trigger negative frequency-dependent selection by predators against the most common morphotype and lead to increased colour and pattern polymorphism in the population."

3) Agree here, I wonder if size should not be in there too?
Our response: Edited the sentence by adding 'differently sized textures' as (Lines 45-46):" Another mechanism generating variability in camouflaged prey could be if camouflage is required to blend into variable backgrounds (e.g., matching differently sized textures).".

4) Sentence here.
Our response: Fixed (as 'at the same time').

5) Word here - organisms
Our response: Fixed (as organisms).

6) So, nocturnal moths are stationary in the daytime, dirunal moths not so, does this not affect the prediction?
Our response: We have clarified the predictions in more detail as (Lines 70-80): "Diel-activity could be related to moth phenotypic variation as selection from visual predators is most important during the daytime, when nocturnal moths are resting on bark or leaves. For camouflaged prey, predation should favour the colours and patterns resembling resting locations, but there would be no a priori prediction of greater variation in colouration across diel-activity traits, as both nocturnal and diurnal moth species experience predation from diurnal birds. For aposematic species, we expect less variation in aposematic diurnal moths than aposematic nocturnal moths under positive frequency-dependent or stabilizing selection. We assume that in low light conditions it is more difficult for predators to perceive colour variation and thus that nocturnal aposematic moths afford more colour variation than diurnal aposematic moths. For the latter, daylight conditions should enhance transmission of colour signals to predators, which can be assumed to select for warning signal stability."

7) I know the details will be elsewhere, but can you indicate how n varies for each species?
Our response: Indeed, the full list of specimens can be found from the supplementary materials. To summarize, the lowest number of samples per species was 14. Only 6 percent of all the species sampled (5/82 species) had fewer samples than 20 per species, 12 percent (10/82 species) had 20-30 samples, and 82 per cent (67/82 species) had at least 30 samples (reported in supplementary material). In more detail, the sample size of how n varies by species is as follows: Anania terrealis 30, Anania verbascalis 30, Evergestis forficalis 30, Scoparia basistrigalis 30, Udea ferrugalis 30, Calliteara pudibunda 50, Colobochyla salicalis 30, Dicallomera fascelina 60, Herminia grisealis 22, Hypena

crassalis 30, Hypena proboscidalis 30, Hypena rostralis 30, Laspeyria flexula 30, Lygephila craccae 30, Lygephila pastinum 30, Macrochilo cribrumalis 27, Minucia lunaris 30, Nudaria mundana 30, Paracolax tristalis 30, Parascotia fuliginaria 30, Schrankia costaestrigalis 30, Schrankia taenialis 30, Scoliopteryx libatrix 30, Zale lunata 28, Biston betularia 150, Biston strataria 110, Chlorissa viridata 30, Hypomecis roboraria 50, Idaea straminata 30, Odontopera bidentata 60, Phigalia pilosaria 80, Scopula immorata 30, Thalera fimbrialis 30, Pero behrensaria 30, Pero macdunnoughi 30, Malacosoma constrictum 30, Acronicta alni 30, Autographa californica 17, Orthosia hibisci 18, Manduca sexta 30, Megalopyge crispata 30, Anania funebris 30, Anania hortulata 30, Elophila icciusalis 30, Pyrausta ostrinalis 30, Pyrausta purpuralis 30, Arachnis picta 15, Arctia caja 50, Arctia plantaginis 80, Arctia villica50, Callimorpha dominula 30, Diacrisia sannio 50, Euplagia quadripunctaria 90, Gnophaela vermiculata 16, Grammia incorrupta 30, Grammia nevadensis 30, Grammia ornata 30, Hypoprepia fucosa 26, Miltochrista miniata 30, Notarctia proxima 30, Phragmatobia fuliginosa 50, Rhyparia purpurata 30, Spilosoma lubricipeda 50, Tyria jacobaeae 30, Virbia ostenta 14, Abraxas grossulariata 85, Arichanna melanaria 39, Chiasmia clathrata 77, Idaea muricata 30, Lomaspilis marginata 50, Lomaspilis opis 30, Lythria purpuraria 24, Pseudopanthera macularia 30, Rheumaptera hastata hastata 30, Rheumaptera hastata nigricens 30, Anarta myrtilli30, Tyta luctuosa 60, Pyromorpha dimidiata 20, Zygaena filipendulae 22, Zygaena lonicerae 27, Zygaena minos 27, Zygaena viciae 23.

8) Sentence here
Our response: Edited as: "Counter to our predictions, we generally did not find evidence that within-species variability in colour traits is different between aposematic vs camouflaged species (Figures 4 & 5), despite the fact that aposematic and camouflaged species differ markedly in mean values of colour traits (see supplementary materials)."

*****Reviewer 2 Remarks to the Author
The author(s) are to be congratulated for taking on such an ambitious project. Overall, the work is well thought through, clearly presented and the results are believable. Most of the (understandable) weaknesses of the study (e.g. lack of UV component, uncertainty over the natural backgrounds of the moth species) have already been highlighted and discussed, and the author(s) have taken a deep dive into what is known about each moth species. I particularly appreciate keeping observations of a given species to within a 500 km radius given the regional variation observed in many aposematic species (e.g. certain burnet moth species). The fact that both forewings and hindwings were considered is also to the author(s) credit. However, I feel that there are some aspects of the paper that could do with improvement. Below I make some comments and pose some questions which I hope will be of use to the author(s) in making an even clearer and better argued paper.
Our response: We thank the referee for thoughtful and supportive feedback! We have carefully followed the road map provided to refine the quality of our manuscript and hope this work will be a worthy contribution to our community.

Statistics: The coefficient of variation works well as a standardized measure of dispersion, but it is a biased measure of the equivalent measure within a population (see Sokal & Rohlf 2012 Biometry 4th Edition page 56). The bias is small for large samples, but "appreciable" for small samples. Specifically, when a variable is normally distributed at population level, a correction factor of (1 + 1/4n) to the sample CV provides the ML estimate of the population-level CV. If all species had the same sample size, then it would be a fixed adjustment of no consequence (all species equally underestimated to the same small degree when n is large). However, since n varies from 30 to 14, it might marginally under-estimate the population-level CV more in the less well represented species compared to better-represented species. So long as certain classes of moth were not especially under-represented it is unlikely to change anything, but perhaps worth bearing in mind.
Our response: Indeed, we rigorously sought to gain enough samples for each species within limits of accessible online collections and within our geographic range restrictions. Only 6 percent of all the species sampled (5/82 species) had fewer samples than 20 per species, 12 percent (10/82 species) had 20-30 samples, and 82 per cent (67/82 species) had at least 30 samples (reported in supplementary material). The sample sizes were not biased among aposematic vs camouflaged comparisons (Pearson's Chi-Squared test = 0.05, df = 1, p = 0.818), altogether the sampling contained 1525 camouflaged and 1535 aposematic moths, both anti-predator strategies had 41 species represented. We also did revisit the analysis and checked the effect sizes using Cohen's f to account get standardized average effects for the independent variables. We report Eta Squared effect values, however, as discussed later in this response.

Moreover, as Sokal & Rohlf (2012) and multiple sources stress, the CV only has meaning for variables measured on the ratio scale i.e. where there is a true zero and the zero refers to the absence. This seems to be the case for the HSV (where 0,0,0 = black) and it would be worth confirming the 0's refer to absences in all the other more complex measures such as those based on granularity.
Our response: In granularity parameters one may get values that are close to small decimal fractions, but these should not be interpreted as 'structural' non occurrences, but true zeros as noted. Since granularity analysis standardises size (through px/mm ratio) and yields isometric pattern values, the pattern metrics are measured in ratio scale with true zeros at low end of range.

Since (a) the three hypotheses are not mutually exclusive (line 13), (b) the predictors were co-linear (aposematic species are more likely to be diurnal, line 189) and (c) one of the stated predictions was based on a diel activity: anti-predator strategy interaction (line 80), I wonder why models were separately fitted with single predictors (strategy, activity, diet) rather than fitting a full model with all predictors, including interactions that have been recognized in advance. That way, we reduce the number of models fitted, we can see whether diel activity explains the CV of species over and above their anti-predator strategy and we can test directly for the interaction that was anticipated. Of course, if diel activity and anti-predator strategy are highly co-linear the predictors may obscure one another's

effects, but this would not be a problem if the variance could be sequentially partitioned and the predictors were entered in different orders.
Our response: The referee is right; it would be ideal to test all possible main effects and interactions in the same model. However, we also felt it was important to control for underlying shared ancestry to ensure that results were independent of that evolutionary history. phyloANOVA does not allow testing interactions in the same model. Instead, it allows testing single predictors while considering phylogenetic parameters. Thus, we have opted to keep phyloANOVA in our analysis as it stands. Regardless of the analysis method, anti-predator strategy was highlighted as being the most significant predictor of phenotypic variables above all.

The red highlighting of high F values in Figure 4 helps draw the eye to big differences between factor levels compared to variation within factor levels but note that the variance ratio is only a crude metric of effect size (the Eta-squared statistic provides a more principled measure). Of course, since the numerator degrees of freedom (and possibly the denominator df) differ for the NHST tests being displayed, the same value F could be considered consistent with the NH for tests involving 2 classes (1 df e.g. anti-predator strategy) than but not for tests involving 4 classes (3 df e.g. diet), say. I assume that the MS that is widely reported in the results refers to the "mean square", but it is not very meaningful in itself (what does it mean if the MS is 40584.73 or 40.5?). It would be better to give an indication of the average effect size and provide the two degrees of freedom associated with the F-ratio.
Our response: We agree that providing effect size measure is useful and we have now included Eta-Squared statistics into results. phyloANOVA statistics, we would like to stress that analysis included Holm-Bonferroni method that controls for potential familywise error rates for multiple hypothesis tests. Thus, the method reduces possibility of type I error when performing multiple comparisons and the reported F-values to our understanding take different levels of comparison into accounts. In any case, we agree that Mean Square metrics may not be intuitive measure of effect here and we have omitted those from the main text. Also, figure 4 is re-drawn using Eta Squared values.

Methods: It is a little unclear to me how the classifications of each moth species were arrived at. In particular, species are listed as aposematic or cryptic in the SI, although the associated summaries often state “No literature on aposematism” or “No literature on camouflage”. How were these classifications made if there was no literature to go on? I may have missed this. If the classification was based on judgement of conspicuousness, who judged it and how? Looking at the images it may have been a clear-cut distinction, but I’ve seen enough moths to know that it isn’t always the case especially given uncertainty over backgrounds. In addition, since some moth species have highly conspicuous hindwings used to startle would-be predators (line 205), I’m guessing that if a personal classification was adopted it was based on forewing colour patterns.
Our response: We have added the following section into methods to clarify (lines 254-262): “For categorization, we sought experimental evidence on whether a species had been documented as having chemical defence, conspicuous appearance and/or was the

subject of avoidance learning by predators (i.e., aposematism). Also, we searched to determine if a species had been shown to* lack *chemical defence or rely chiefly on background matching (i.e., camouflage). Information about the categorization is found from tables 2-4 in supplementary material. When species lacked these data, we searched for evidence on closely related species and consulted an expert who subjectively decided the category based on moths' appearance guided by forewing coloration. We did not include species that may use startle in our sampling (e.g., we left Catocala moths that have cryptic forewings and conspicuous hindwings away)".

We see moth species listed as monophagous, mycophagous, oligophagous or polyphagous. However, adult moths (which have probiscis) often feed (if at all) in different places and on very different resources (nectar, sap, fruit) than their larvae (which have mandibles). It seems to me that in the context of the hypotheses proposed to explain wing pattern variation, the diet that matters is that of the adult not the larvae. Unfortunately I cannot find any mention of the life stage to which the diet refers. Naturally some moths complete their life cycle close to their host plant so arguably larval diet can say something about adult plant associations - but I don't see any arguments of this nature in the text. Arguably, it could be a reason why the authors don't see a strong diet specialization effect! The classification "mycophagous" is also an odd one as it doesn't refer to diet breadth per se (can you be a mycophagous specialist or generalist?); a sentence to justify it would therefore help. Finally, larval monophagy is often difficult to pin down – indeed, several of the species listed a monophagous in Table S2 have been found on closely related host plant species. In short, more details are needed as to how the classifications were arrived at.

Our response: Very good points indeed! We have clarified that "by diet" we refer to larval diet here. The section describing classifications has been edited as (lines 264-274): "Regarding autecological attributes of these species, for diel-activity (3 factor levels), 18 species were cathemeral, 15 diurnal and 49 nocturnal. For larval dietary-preference (4 factor levels), 12 species were monophagous herbivores (feed on a single genus of host), 14 oligophagous herbivores (feed on more than one genus in the same family) and 51 polyphagous herbivores (feed on more than one plant family) and 5 mycophagous (feed on fungi). Some moths complete their life cycle close to their host plants and thus, larval dietary niche may predict phenotypic associations in adults. We also justify using larval diets, because they may allow sequestration of toxic chemicals, which could be related to both aposematism and dietary specialization, if sequestration requires adaptations to specific compounds (e.g., monarch butterflies). Less is known about moth adult feeding, but it tends to be more generalized as moths are often nectar feeders. With regard to 'mycophagous', these species could plausibly be specialist or generalists. See supplementary materials for references (Table S2)."

Line 23 (and elsewhere) is it a truly a "hypothesis" that camouflaged moth species are more phenotypically variable than aposematic species or a prediction? Is it not a prediction that arises from a broader belief? Surely the hypothesis is that predatory behaviour to deal with an anti-predator strategy generates a form of frequency-dependence which either promotes

diversity (search image) or selects against it (avoidance learning)? If true, it makes a certain predictions (which can be tested) regarding the degree of intra-specific variation in species that employ different anti-predator strategies strategies.
Our response: Agreed, we have clarified tone more towards 'predictions' as suggested.

Line 66 AT the same time.
Our response: Fixed.

Line 66 Arguably the regional polymorphism does "fit ..the classic paradigm" since it is entirely consistent with positive frequency selection (strength in numbers).
Our response: Correct. We have modified the sentence as follows: "Thus, aposematic prey are repeatedly shown to be locally under positive frequency-dependent selection and under stabilizing selection for uniform signals".

*****Reviewer 3 Remarks to the Author
In this MS, the authors examined how antipredator strategy, activity, and diet influence intraspecific pattern and color variability among Lepidopterans. They found that antipredator strategy was the strongest predictor of pattern variability (aposematic species were less variable than camouflaged species), nocturnal species were more variable than cathemeral or diurnal species, and there were no effects of any covariate on color variation. Overall I found the paper interesting and the results mostly intuitive. My general criticisms on the paper are that there are many instances where the authors need to provide more clarity or exposition as it often reads as though it were written for a more specialist journal where readers have strong background knowledge of Lepidopterans and the deep literature on the evolution of animal coloration (not a Nature journal). Second, there were obvious grammatical issues throughout the manuscript so the paper needs a very thorough revision to correct these errors.
Our response: We thank the referee of thoughtful feedback. We have carefully considered all comments related. The manuscript has been read thoroughly and revised again by a native English speaker. We consider these changes have significantly helped us to increase fluency to general readership and thus higher impact for the community.

Comments: L2: The start of the abstract is too abrupt/specific - it should be more general about coloration evolution and aposematic and camouflage strategies.
Our response: We have included an opening sentence (Lines 2-3): "Natural selection generally favours phenotypic variability in camouflaged organisms, whereas aposematic organisms are expected to evolve a more uniform warning coloration.". In addition, we have shortened the abstract to make it more concise and increase the clarity.

L37: Citations needed to support this sentence.
Our response: Added.

L50-53: I'm not sure I would call this relaxed selection. Relaxed selection implies that there was once strong selection for something and then that lessened. What the authors describe here is that there is simply general selection for particular colors and pattern elements, but weak overall selection (i.e., not stabilizing) governing how those colors/pattern elements come together.

Our response: We have modified this according to referee's suggestion as follows (Lines 45-49): "Another mechanism generating variability in camouflaged prey could be if camouflage is required to blend into variable backgrounds (e.g., matching differently sized textures). Prey might be more variable as they are under weak selection to match a variety of backgrounds. This variability in part may result from an increase of rare alleles by genetic drift, rather than due to strict negative frequency-dependent selection".

L79-81: Explain why - this isn't intuitive to me.

Our response: Further clarified as (Lines 75-81): "For aposematic species, we expect less variation in aposematic diurnal moths than aposematic nocturnal moths under positive frequency-dependent or stabilizing selection. We assume that in low light conditions it is more difficult for predators to perceive the colour variation and we may expect that nocturnal aposematic moths afford more colour variation than diurnal aposematic moths. For the latter, daylight conditions should enhance transmission of colour signals to predators, which can be assumed to select for warning signal stability".

Results: Since the F values are in the Figure, they aren't required to report in the results. It's also not evident what MS is. This section can be shortened if more of these stat results are put into the figure.

Our response: Edited as suggested. MS refers to 'mean square'. We have removed the MS values from the main text as suggested by another reviewer to shorten the results. We report F-values but offer test statistics for effect size in the figure 4.

L267: please describe the standardized way that images were taken - list all of the criteria used.

Our response: We have included more description about the standardised way how museums often take the images as follows (lines 216-218):" for objective analysis museum photos should prioritise standard photographic conditions, uncompressed file types, diffused natural light source, grey standards as well as scales."

However, there is no universally accepted standard for taking images that museums use. The methods should always include a) a photographic standard (of known reflectance), b) obtaining well-focused and well-lit images (in-focus with diffuse natural lighting) of good quality samples (retrievable in raw image file format) and c) retrievable sampling information (data on where and when samples were collected). Here, we have limited our sampling to those that follow objective image digitalisation: poor-quality images in any way were not considered.

L302: I don't understand why images for a species were all selected from a single geographic location. If you want to examine species variability, wouldn't you want to gather images of that species from across its range to indicate intraspecific variation??
Our response: To answer our study question, it was necessary to select specimens within the range that we can assume largely the same predator species are preying upon the given moth population while minimizing shifts in climatic conditions that may result in shifting color or pattern of moths due to thermoregulation, for instance. We have clarified this in the methods as (lines 511-519):" Individuals representing a species were chosen from collections within a 500 km radius geographical proximity to one another (as sampling must include a decent range ecologically but must also minimize the influence of climate and other ecological differences –like changes in predator communities– on colouration), and that had digital images.

L318-323: Please define these more clearly, especially granularity. It is not something that's known to people that don't specialize in coloration. What is dominance??? The granularity parameters need to be clearly defined and listed before they are discussed in the results.
Our response: We have added further detail on pattern metrics. To extract pattern information, we applied a pattern analysis technique (a 'granularity' analysis) involved decomposing an image into a series of spatial frequencies using Fourier analysis and band pass filtering, followed by determining the relative contribution of different marking sizes to the overall pattern (Barbosa et al. 2008, Hanlon et al. 2009, Stoddard and Stevens 2010). For the pattern data, we used dominance (i.e., maximum pixel energy – the energy at the spatial frequency with the highest pixel energy), marking size (i.e., the spatial frequency with peak energy) and contrast (i.e., summed power or amplitude – the energy summed across all scales). These key metrics are analogous to wave characterizations in physics. Pattern analysis was conducted in custom files for Image J (Troscianko and Stevens 2015). The method is established and has been thoroughly documented (www.empiricalimaging.com) along with the pattern ('granularity') analysis (http://www.empiricalimaging.com/knowledge-base/pattern-energy-granularity-analysis/).

The methods now stand as (Lines 365-380): "To extract pattern information, we applied a pattern ('granularity') analysis technique that decomposes an image into a series of spatial frequencies using Fourier analysis and band pass filtering and follows with determining the relative contribution of different marking sizes to the overall pattern (Barbosa et al. 2008, Hanlon et al. 2009, Stoddard and Stevens 2010). Granularity analysis is a powerful tool for quantifying animal patterning as it objectively measures variation in phenotypic appearance in terms of arrangement in luminance composition (Barbosa et al. 2008, Hanlon et al. 2009, Stoddard and Stevens 2010, Troscianko and Stevens 2015). The analysis was conducted by setting linear increments of one pixel from 1 to 100 pixels corresponding to tenth of a millimetre increments up to 1 centimeter. This method controls for size variation and scales wings by a known pixel

/ millimetre ratio, which standardises size and thus yields isometric pattern values. We used 20 luminance bands range in pattern analyses. We used maxPower (i.e., dominance – the energy at the spatial frequency with the highest pixel energy), maxFreq (i.e., marking size – the spatial frequency with peak energy) and sumPower (i.e., contrast – the energy summed across all scales) of the micaToolbox pattern variables which here are considered as pattern dominance, marking size (or sizing) and contrast, respectively (Figure S1B). Pattern analysis was conducted in custom files for Image J (Troscianko and Stevens 2015).".

Figure 4: Change "strategy" to antipred or ap-strategy
Our response: Done, changed to 'antipred'.

< end of comments >

REVIEWERS' COMMENTS

Reviewer #1 (Remarks to the Author):

1) Issue of size. I thank the authors for taking this on, and reporting the non-significant effect.

2) Search images & similar & Clarity over FDS - the text is now improved in this respect (at least in my view!).

3) Diurnal/Nocturnal - this is now improved, thanks to the authors

4) sample sizes - thank you for adding this information.

Reviewer 2 - statistical issues, best dealt with by them; but it strikes me that the authors have made good efforts to resolve their issues.

I must apologise to the authors for my delay in dealing with this MS, a bout of Covid, and some other unusual circumstances left me with a significant backlog - I do apologise.

Reviewer #2 (Remarks to the Author):

I have reviewed the revised manuscript "Predator selection on phenotypic variability of cryptic and aposematic moths". Overall, I think that the authors have done a good job of responding to my earlier comments (notably: bias in estimating population CV from small samples, the possibility of fitting a single model to evaluate predictions simultaneously, using effect sizes rather than F or MS, questions as to how classifications of moths were derived and whether the dietary range referred to adults or larvae). On re-reading, I only have minor comments.

1. Line 141 and following: It would help to specify the two degrees of freedom associated with each F-ratio.
2. Line 158. In what way were these results noteworthy? The associated Pr(>Statistic|NH) values appear unremarkable, but perhaps there is some biology I have overlooked.
3. The term "taxa" is used repeatedly in the abstract, methods and significance statement (e.g. "82 moth taxa"), but the predictions and results use the term "species" (e.g. "41 aposematic species"). I appreciate that the species status of some of these moths may not be known. However, I think it would help to be consistent, or at least justify the use of two terms - because otherwise readers may begin to wonder whether they have missed something.
4. Line 404. RStudio is simply the Integrated Development Environment (IDE) for R. The phytools package is implemented in the R programming language, which is much more important to know.

Reviewer #3 (Remarks to the Author):

The authors have done a fine job addressing my minor concerns from the previous draft. My only suggestion is that the new paper on variable aposematic patterning in striped skunks by Walker et al (Evolution) could be added to the introduction in line 65 as it is from a unique taxon relative to most aposematic studies.

(NCOMMS-23-16093)
*****Reviewer #1 (Remarks to the Author):
1) Issue of size. I thank the authors for taking this on, and reporting the non-significant effect. 2) Search images & similar & Clarity over FDS - the text is now improved in this respect (at least in my view!). 3) Diurnal/Nocturnal - this is now improved, thanks to the authors 4) sample sizes - thank you for adding this information.
Our response: We thank the referee for the positive response towards our manuscript.

*****Reviewer #2 (Remarks to the Author):
On re-reading, I only have minor comments.
1. Line 141 and following: It would help to specify the two degrees of freedom associated with each F-ratio.
Our response: We thank the referee for the comment. However, the degrees of freedom are not generated along the calculation of phylANOVA test statistics as in classical parametric tests. Instead, the method relies on the simulated null distribution for comparisons and therefore the degrees of freedom cannot be explicitly reported.

2. Line 158. In what way were these results noteworthy? The associated Pr(>Statistic|NH) values appear unremarkable, but perhaps there is some biology I have overlooked.
Our response: We have revised the sentence as:" Albeit a statistically non-significant observation, aposematic moths show a little more variability in colour than camouflaged moths in hindwings".

3. The term "taxa" is used repeatedly in the abstract, methods and significance statement (e.g. "82 moth taxa"), but the predictions and results use the term "species" (e.g. "41 aposematic species"). I appreciate that the species status of some of these moths may not be known. However, I think it would help to be consistent, or at least justify the use of two terms - because otherwise readers may begin to wonder whether they have missed something.
Our response: Revised as suggested. Also, we have added a clarification in the beginning of methods: "We use the term species for consistency although the species status of some of these moth taxa may not be clear.".

4. Line 404. RStudio is simply the Integrated Development Environment (IDE) for R. The phytools package is implemented in the R programming language, which is much more important to know.
Our response: Revised as suggested: "implemented using the phytools package (Revell 2012) in the R programming language".

*****Reviewer #3 (Remarks to the Author):
The authors have done a fine job addressing my minor concerns from the previous draft. My only suggestion is that the new paper on variable aposematic patterning in striped skunks by Walker et al (Evolution) could be added to the introduction in line 65 as it is from a unique taxon relative to most aposematic studies.
Our response: Agreed. We have included the Walker et al. reference as suggested.
< end of comments >
